# Supplementary material for: The C5a/C5a receptor 1 axis controls tissue neovascularization through CXCL4 release from platelets
Source: Nat Commun. 2021 Jun 7;12:3352. doi: 10.1038/s41467-021-23499-w (PMC8185003; doi:10.1038/s41467-021-23499-w)
Supplement: Supplementary file 1 — Supplementary Information [file 41467_2021_23499_MOESM1_ESM.pdf]

Supplementary information

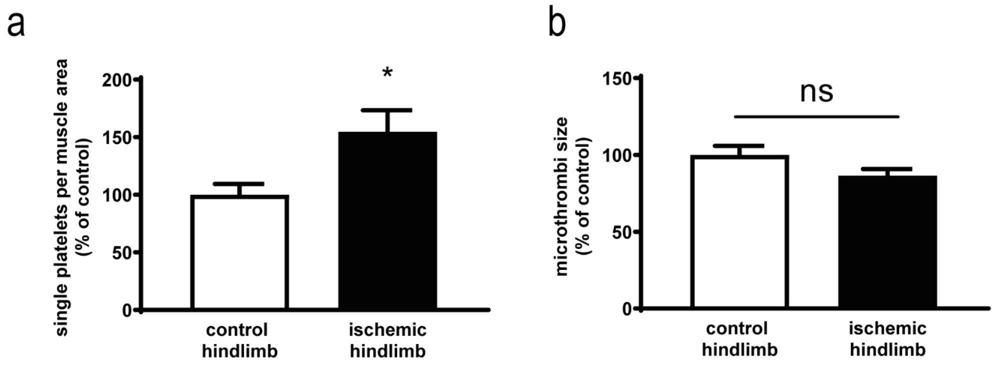

Supplementary figure 1: Analysis of platelet microthrombi distribution in ischemic vs. nonischemic hindlimb tissue

(a) CD42b-positive single platelets were quantified by size characteristics using automated digital image analysis as described in the Methods part in whole muscle sections. There was a significantly higher number of platelets within ischemic versus nonischemic hindlimbs of mice. Data are shown as the mean±SEM (n=6 whole muscle sections per group) and are displayed as % of control. The readings in nonischemic hindlimbs represent 100% in both graphs. *p<0.05. (b) In nonischemic control hindlimbs, microthrombi were slightly larger than in ischemic hindlimbs but this difference was not statistically significant. Data are shown as the mean±SEM (n=11-12 whole muscle sections per group) and are displayed as % of control. The readings in nonischemic hindlimbs represent 100% as expressed as size in pixels in both graphs. ns = not significantly different. Student's t-test in (a), (b).

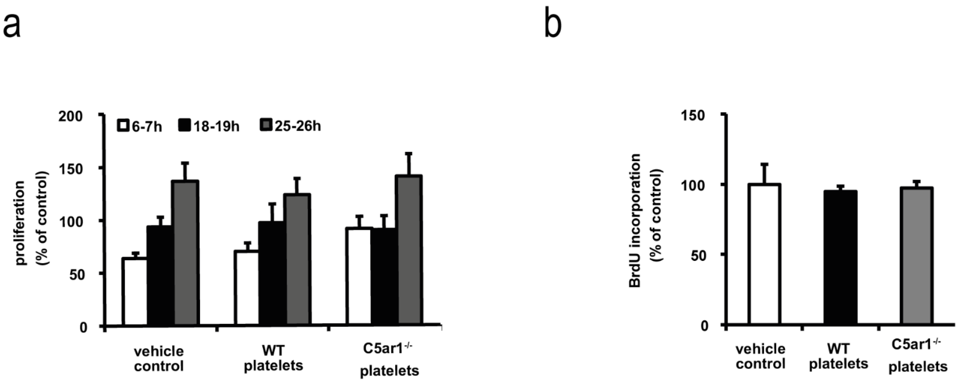

Supplementary figure 2: The platelet C5aR1 receptor has no impact on endothelial proliferation
(a) The coincubation of washed platelets isolated from WT or *C5ar1*$^{-/-}$ mice with endothelial cells (MHEC-5T) did not alter endothelial proliferation after 6, 18 and 25 h. Data are displayed as the mean±SEM (n=5 independent experiments). The number of cells at t0 represents 100%. (b) The effect of platelet C5aR1 on endothelial proliferation of MHEC-5T cells was also assessed using a BrdU-incorporation ELISA after 18 h. Data are displayed as the mean±SEM (n=5 independent experiments). The BrdU-incorporation measurement of cells stimulated with vehicle control represents 100%. Two-way ANOVA with Bonferroni's post hoc test in (a) and (b).

a

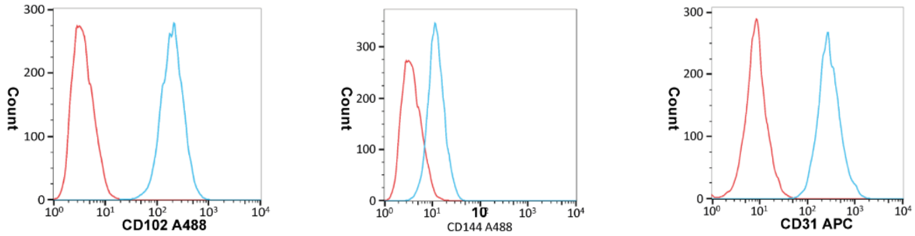

b

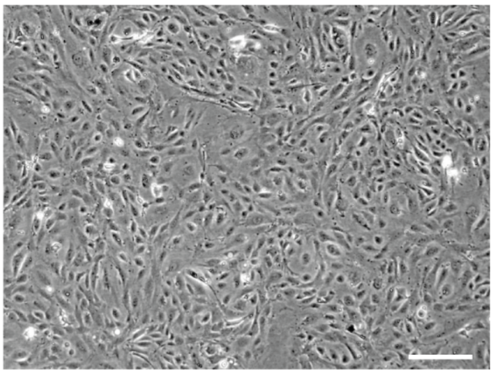

Supplementary figure 3: Isolation and cell purity of primary mouse lung endothelial cells (MLECs)

Primary mouse endothelial cells were isolated from the lungs of C57BL/6 mice following a multistep protocol as described in the Methods section. (a) For assessment of cell purity, MLECs were stained for CD102, CD144 and CD31. Depicted are representative flow cytometric analyses for these markers; red curves show isotype control, blue curves specific staining. Cell preparations were only used for experiments if the purity of cells was at least 90%. Cells positive for all three markers were considered endothelial cells. (b) MLECs plated into gelatin-coated cell culture dishes displayed the typical cobblestone pattern in culture. Image is representative of at least 10 images acquired. Scale bar represents 200 µm.

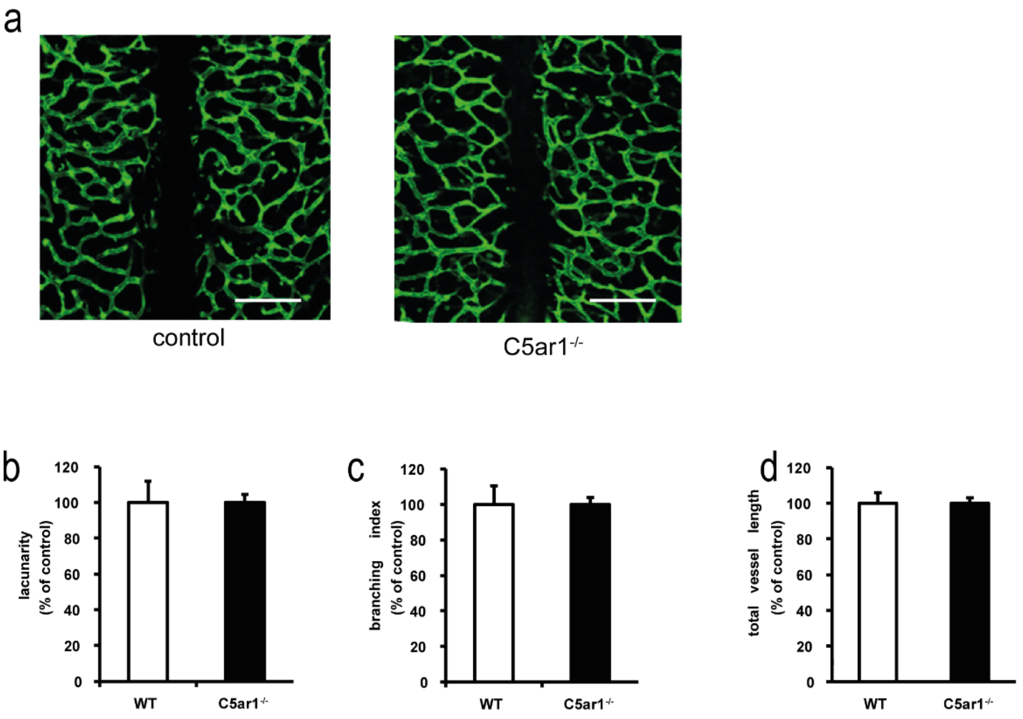

Supplementary Fig. 4: C5aR1-deficient mice do not display altered developmental angiogenesis

Angiogenesis was evaluated in the embryonic hindbrains of WT and *C5ar1*$^{-/-}$ mice. (a) Developmental angiogenesis was quantified at day E11.5 using IB4 to stain endothelial cells in hindbrain whole-mounts (n=6). Differences were not observed between WT and *C5ar1*$^{-/-}$ ventricular plexuses. Scale bars represent 200 µm. Accordingly, differences were not observed in average lacunarity (b), the number of junctions per area (branching index; c) or the total vessel length per analyzed area (d). Data are shown as the mean±SEM (n=6 wholemounts analyzed per group) Student's t-test in (b) - (d).

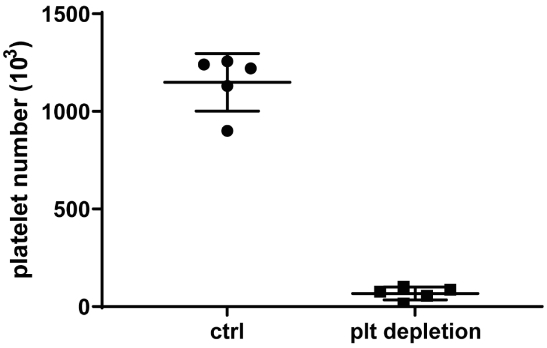

Supplementary figure 5: Monitoring of successful platelet depletion
For some experiments, mice were injected with platelet depleting serum (10 µl per dose in 200 µl PBS, Accurate Chemical and Scientific, Westbury, USA). To monitor successful platelet depletion, blood was drawn retroorbitally from WT mice and platelet count was determined using a Sysmex cytometer. Platelet depleting serum induced a drop in platelet count > 90%. Data are shown as the mean±SEM (n=5 animals per group). Student's t-test.

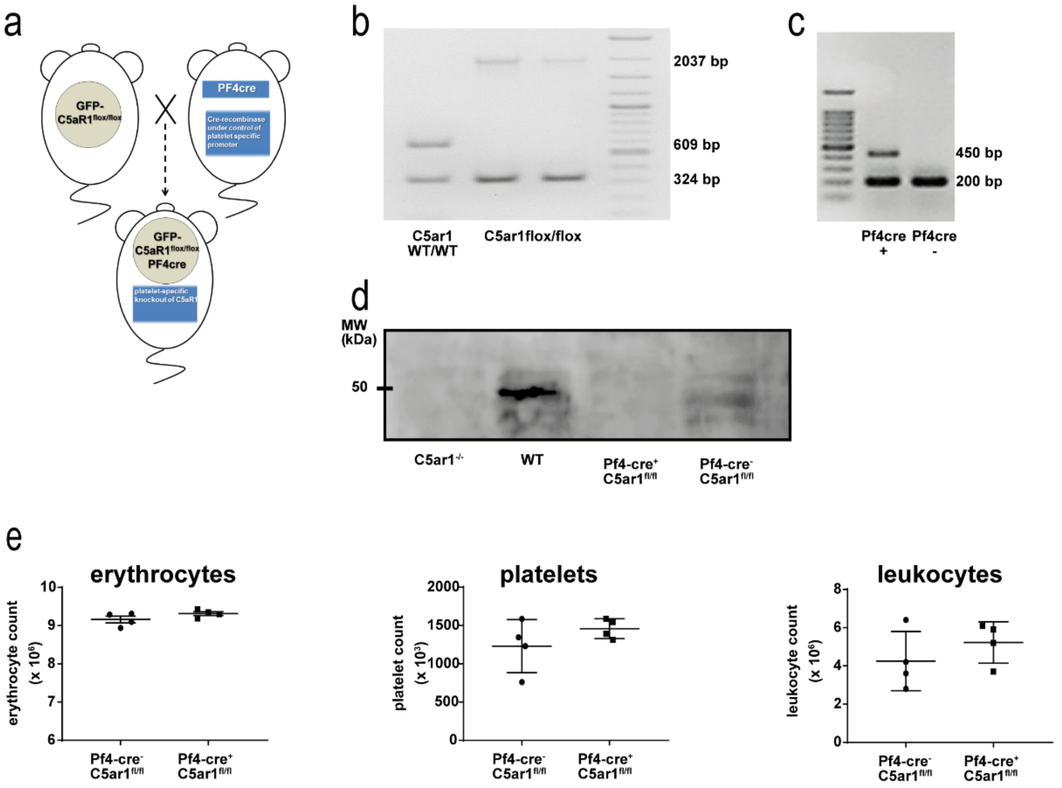

Supplementary figure 6: Generation and characterization of platelet-specific C5aR1-deficient mice

(a) Platelet-specific *C5ar1*-knockout mice were generated by crossing GFP-*C5ar1*$^{fl/fl}$ with *Pf4-cre*$^{+}$ mice. (b) Genotyping of *Pf4-cre* GFP-*C5ar1f*lox mice was performed by amplification of a product of 2037 base pairs (bp) specific for the floxed *C5ar1* insert. C5aR1 WT mice were identified by amplification of a product of 600 bp. (c) PCR for detection of PF4-dependent Cre-recombinase was performed in DNA samples from the same animals. Cre-positivity is indicated by amplification of a product of 450 bp.

(d) Western blot analysis of C5aR1 expression in platelet lysates from *C5ar1*$^{-/-}$, WT, *Pf4-cre*$^{+}$ GFP-*C5ar1*$^{fl/fl}$ and Pf4-cre^{-} GFP-*C5ar1*$^{fl/fl}$ mice revealed the absence of C5aR1 in *C5ar1*$^{-/-}$ and *Pf4-cre*$^{+}$ GFP-*C5ar1*$^{fl/fl}$ mice, or the presence of C5aR1 in WT and *Pf4-cre*$^{-}$ GFP-*C5ar1*$^{fl/fl}$ platelets. One of 4 independent experiments is depicted. (e) There was no significant difference in red blood cell, platelet or leukocyte count between *Pf4-cre*$^{+}$ GFP-*C5ar1*$^{fl/fl}$ and *Pf4-cre*$^{-}$ GFP-*C5ar1*$^{fl/fl}$ mice. Data are shown as the mean±SEM (n=4 animals per group). Student's t-test in (e).

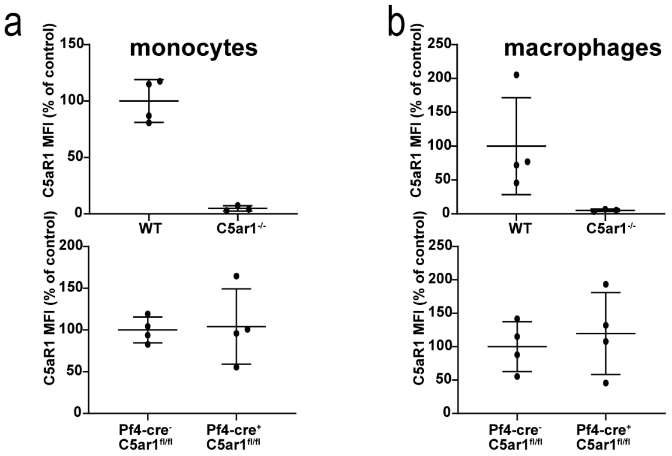

Supplementary figure 7: Characterization of C5aR1 expression on monocytes and macrophages in platelet-specific C5aR1-deficient mice

Blood was drawn from WT, C5aR$^{-/-}$, and *Pf4-cre*$^{-}$ GFP-*C5ar1*$^{fl/fl}$ and *Pf4-cre*$^{+}$ GFP-*C5ar1*$^{fl/fl}$ mice. Using flow cytometry, the expression of C5aR1 was assessed in whole blood on (a) monocytes and (b) macrophages. For the gating antibodies used please refer to Methods. Data are shown as the mean±SEM (n=4 animals per group) and as % of control. The MFI measurement for C5aR1 in blood from WT mice or *Pf4-cre*$^{-}$ GFP-*C5ar1*$^{fl/fl}$ represent 100%. Student's t-test in (a), (b).

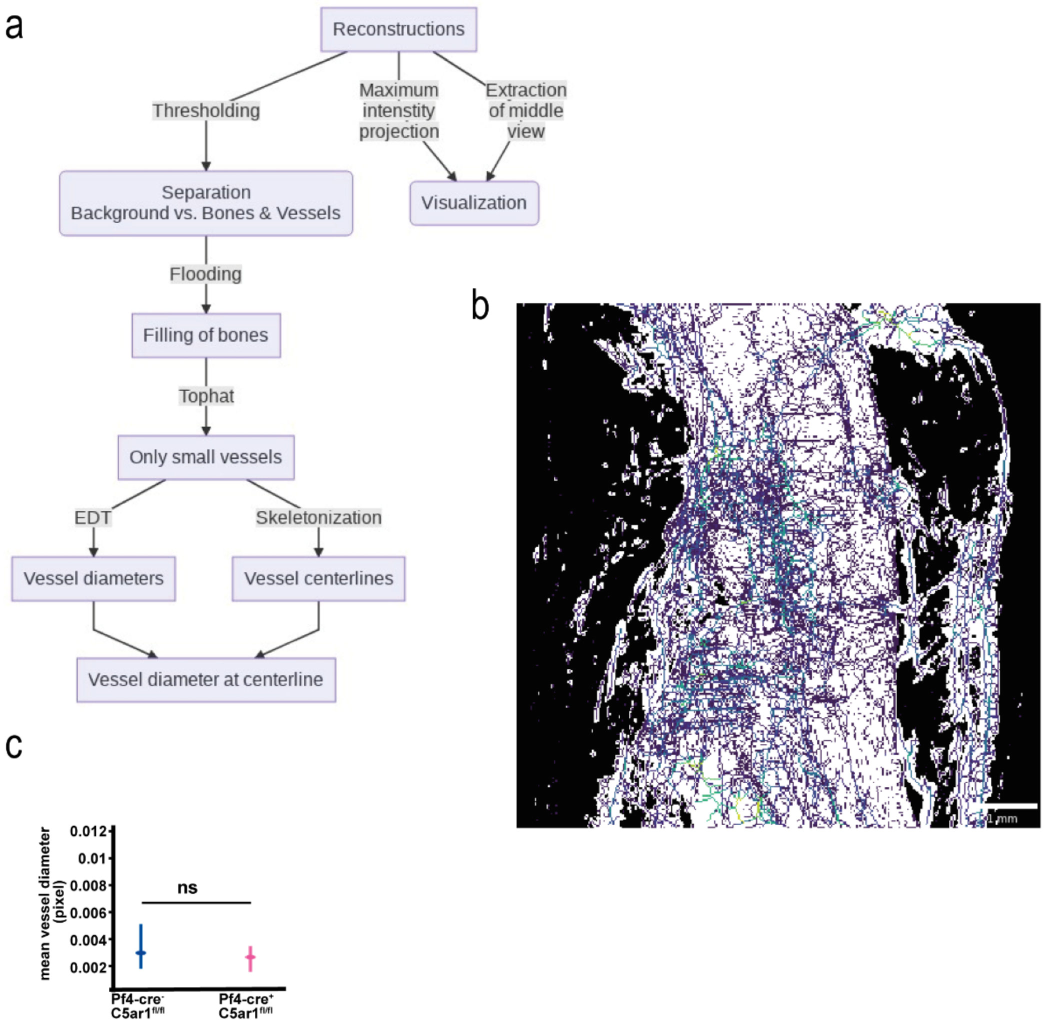

Supplementary figure 8: Schematic representation of the algorithm, how the vessel trees and vascular characteristics of *Pf4-cre*$^-$ GFP-*C5ar1*$^{fl/fl}$ and *Pf4-cre*$^+$ GFP-*C5ar1*$^{fl/fl}$ mice were analyzed by microCT.

(a) Flow chart of analysis algorithm of microCT quantification. (b) Representative image of skeletonized vessel tree in coronal projection. The scale bare represents 1 mm. (c) Vessel trees in nonischemic limbs of *Pf4-cre*$^-$ GFP-*C5ar1*$^{fl/fl}$ and *Pf4-cre*$^+$ GFP-*C5ar1*$^{fl/fl}$ mice were analyzed. There is no significant difference in mean vessel diameter in nonischemic hindlimbs proximal to the knee in between *Pf4-cre*$^-$ GFP-*C5ar1*$^{fl/fl}$ and *Pf4-cre*$^+$ GFP-*C5ar1*$^{fl/fl}$ mice**.** Data are shown as a mean ±95% confidence interval (n=3-4 animals i.e. vessel trees per group). Data is stated in pixels/voxels and 1 voxel represents 21.6 um. Student's t-test in (c).

Number of families	1				
Number of comparisons per family	52				
Alpha	0.05				
Bonferroni's multiple comparisons test	Mean Diff.	95.00% CI of diff.	Significant?	Summary	Adjusted P Value
CXCL4 vs. ADAMTS1	8168	2892 to 13443	Yes	****	<0.0001
CXCL4 vs. Amphiregulin	6649	1373 to 11924	Yes	**	0.002
CXCL4 vs. Angiogenin	6738	1462 to 12014	Yes	**	0.0016
CXCL4 vs. Angiopoietin-1	1900	-3376 to 7176	No	ns	>0.9999
CXCL4 vs. Angiopoietin-3	9130	3854 to 14405	Yes	****	<0.0001
CXCL4 vs. Coagulation Factor III	8814	3538 to 14089	Yes	****	<0.0001
CXCL4 vs. CXCL16	9006	3730 to 14281	Yes	****	<0.0001
CXCL4 vs. Cyr61	8150	2874 to 13426	Yes	****	<0.0001
CXCL4 vs. DLL4	7191	1915 to 12467	Yes	***	0.0005
CXCL4 vs. DPPIV	5882	606.3 to 11158	Yes	*	0.0128
CXCL4 vs. EGF	7193	1917 to 12468	Yes	***	0.0005
CXCL4 vs. Endoglin	5576	299.8 to 10851	Yes	*	0.0258
CXCL4 vs. Endostatin/Collagen XVIII	4974	-301.7 to 10250	No	ns	0.0945
CXCL4 vs. Endothlin-1	6955	1679 to 12230	Yes	***	0.0009
CXCL4 vs. FGF acidic	7271	1995 to 12546	Yes	***	0.0004
CXCL4 vs. FGF basic	7238	1962 to 12513	Yes	***	0.0004
CXCL4 vs. KGF	8239	2963 to 13514	Yes	****	<0.0001
CXCL4 vs. Fractalkine	7555	2279 to 12830	Yes	***	0.0002
CXCL4 vs. GM-CSF	6554	1278 to 11829	Yes	**	0.0025
CXCL4 vs. HB_EGF	6454	1178 to 11729	Yes	**	0.0032
CXCL4 vs. HGF	6280	1004 to 11556	Yes	**	0.0049
CXCL4 vs. IGFBP-1	5636	360.0 to 10911	Yes	*	0.0226
CXCL4 vs. IGFBP-2	6473	1197 to 11749	Yes	**	0.0031
CXCL4 vs. IGFBP-3	7483	2208 to 12759	Yes	***	0.0002
CXCL4 vs. IL-1a	7750	2474 to 13025	Yes	***	0.0001
CXCL4 vs. IL-1ß	8467	3191 to 13742	Yes	****	<0.0001
CXCL4 vs. IL-10	8444	3168 to 13719	Yes	****	<0.0001
CXCL4 vs. IP-10	7959	2683 to 13234	Yes	****	<0.0001
CXCL4 vs. KC	6974	1698 to 12250	Yes	***	0.0008
CXCL4 vs. Leptin	6449	1173 to 11724	Yes	**	0.0033
CXCL4 vs. MCP-1	6609	1333 to 11884	Yes	**	0.0022
CXCL4 vs. MIP-1a	6506	1230 to 11781	Yes	**	0.0028
CXCL4 vs. MMP-3	6793	1518 to 12069	Yes	**	0.0014
CXCL4 vs. MMP-8	7268	1992 to 12544	Yes	***	0.0004
CXCL4 vs. MMP-9	7488	2212 to 12763	Yes	***	0.0002
CXCL4 vs. NOV	7969	2693 to 13244	Yes	****	<0.0001
CXCL4 vs. Opsteopoetin	8492	3216 to 13767	Yes	****	<0.0001
CXCL4 vs. PD-ECGF	8192	2917 to 13468	Yes	****	<0.0001
CXCL4 vs. PDGF--AA	6920	1645 to 12196	Yes	***	0.001
CXCL4 vs. PDGF-AB/PDGF-BB	7589	2314 to 12865	Yes	***	0.0002
CXCL4 vs. Pentraxin-3	7250	1974 to 12526	Yes	***	0.0004
CXCL4 vs. PIGF-2	8206	2931 to 13482	Yes	****	<0.0001
CXCL4 vs. Prolactin	7999	2723 to 13274	Yes	****	<0.0001
CXCL4 vs. Proliferin	8293	3017 to 13568	Yes	****	<0.0001
CXCL4 vs. SDF-1	5316	40.50 to 10592	Yes	*	0.0458
CXCL4 vs. Serpin E1	7915	2639 to 13190	Yes	****	<0.0001
CXCL4 vs. Serpin F1	7599	2324 to 12875	Yes	***	0.0002
CXCL4 vs. Thrombosponin-2	7881	2605 to 13156	Yes	****	<0.0001
CXCL4 vs. TIMP-1	7837	2561 to 13112	Yes	****	<0.0001
CXCL4 vs. TIMP-4	7668	2392 to 12944	Yes	***	0.0001
CXCL4 vs. VEGF	8717	3442 to 13993	Yes	****	<0.0001
CXCL4 vs. VEGF-B	8654	3378 to 13929	Yes	****	<0.0001

Supplementary figure 9: Bonferroni analysis of the proteome profiler angiogenesis array

(a) Freshly isolated washed murine platelets were stimulated with C5a for 10 min at 37°C, platelet supernatant was harvested and then subjected to the Proteome Profiler Mouse Angiogenesis Array (R&D, Minneapolis, MN, USA). The array results were quantified as intensity values of supernatant stimulated with vehicle control versus C5a. C5a-induced changes were analyzed by ANOVA with post-hoc Bonferroni analysis. The results of this analysis are represented. C5a-induced CXCL4 secretion reached significance over all other factors except endostatin and angiopoietin-1.

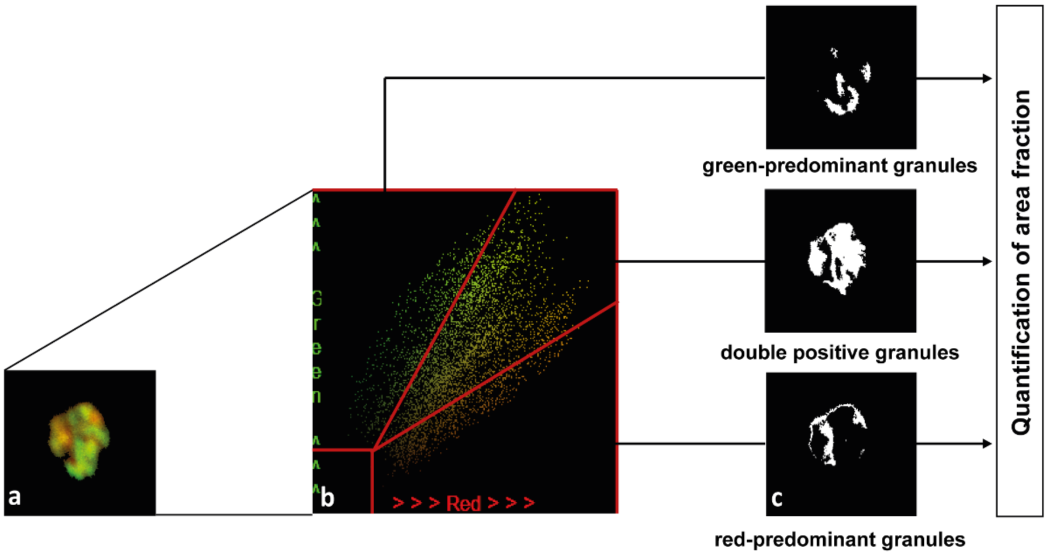

Supplementary figure 10: Platelet granule area fraction assessment
In order to characterize the granule composition of platelets in an objective fashion, single platelets were extracted from confocal immunofluorescence images using Image Pro Plus Software (a). In order to distinguish predominantly red from predominantly green or double positive granules, regions of interest were applied to the fluorescence scatter plot generated by Image Pro Plus (b). Finally, masks were created for each category (c; predominantly red (CXCL4), predominantly green (P-selectin) or double positive) and area fraction was quantified per platelet size.

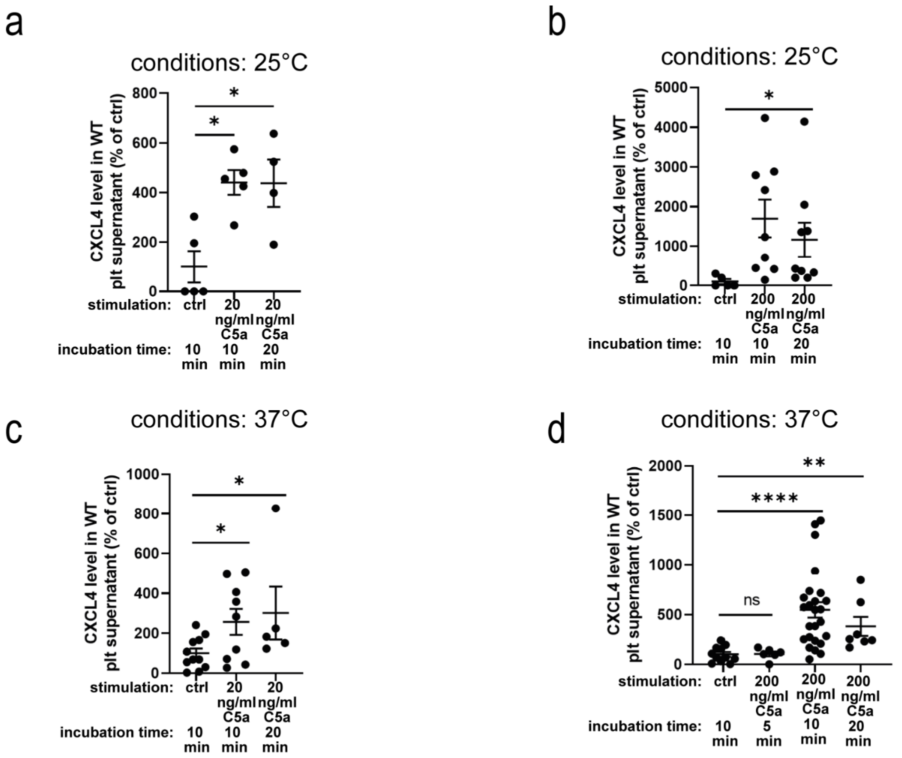

Supplementary figure 11: Time dependency of CXCL4 secretion under different conditions of platelet stimulation.

(a) – (d) Washed murine WT platelets were stimulated with C5a at different concentrations and conditions for different periods of time. The supernatant was analyzed by ELISA. Both at 25°C as well as 37°C, optimal CXCL4 secretion was achieved after 10 minutes of stimulation. Data are shown as the mean±SEM (n=4-25 independent experiments) of CXCL4 level measured by ELISA in platelet supernatant and are displayed as % of control. The CXCL4 protein level of vehicle-stimulated platelet supernatant represents 100% in all graphs. *p<0.05. One-way ANOVA with Bonferroni post-hoc correction in (a) – (d).

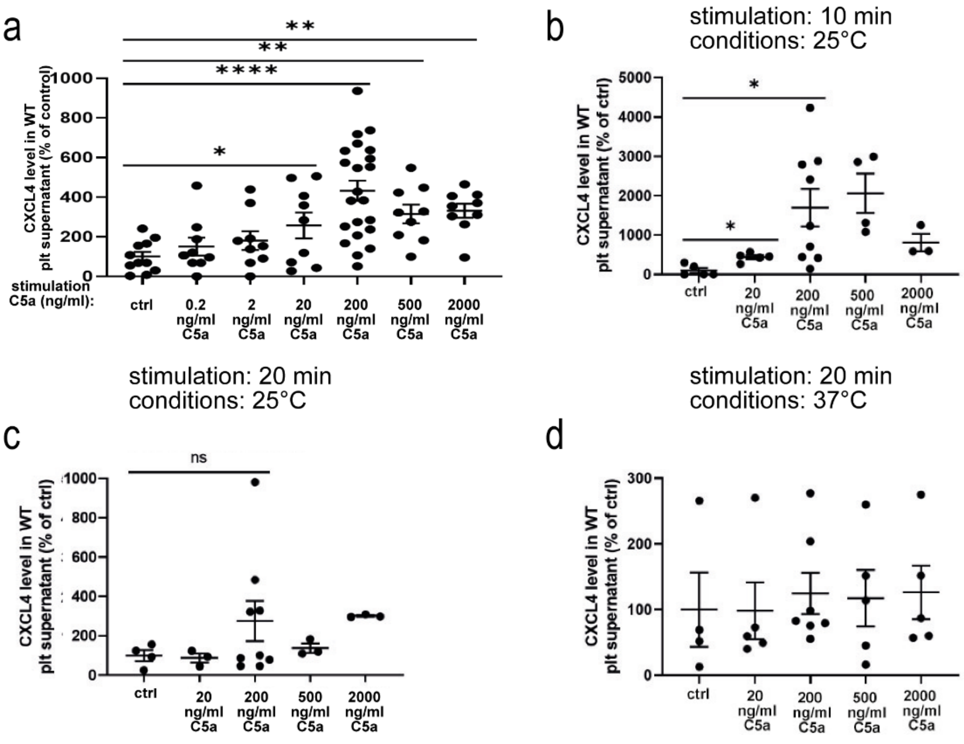

Supplementary figure 12: C5a dose dependently induces CXCL4 secretion from platelets under different stimulation conditions.

Washed murine WT platelets were stimulated with different concentrations of C5a at different conditions. The supernatant was analyzed by ELISA. Both at 37°C (a) and 25°C (b), 10 minutes stimulation induced dose-dependent CXCL4 secretion upon C5a stimulation. Maximum CXCL4 release is reached at a C5a concentration of 200 ng/ml. After 20 minutes, particularly at 37°C (d), no significant upregulation of CXCL4 was observed any more, as baseline CXCL4 secretion increased. In (a) – (d) data are shown as the mean±SEM (n=4-25 independent experiments) of CXCL4 level measured by ELISA in platelet supernatant and are displayed as % of control. The CXCL4 protein level of vehicle-stimulated platelet supernatant represents 100% in all graphs. *p<0.05. One-way ANOVA with Bonferroni post-hoc correction in (a) – (d).

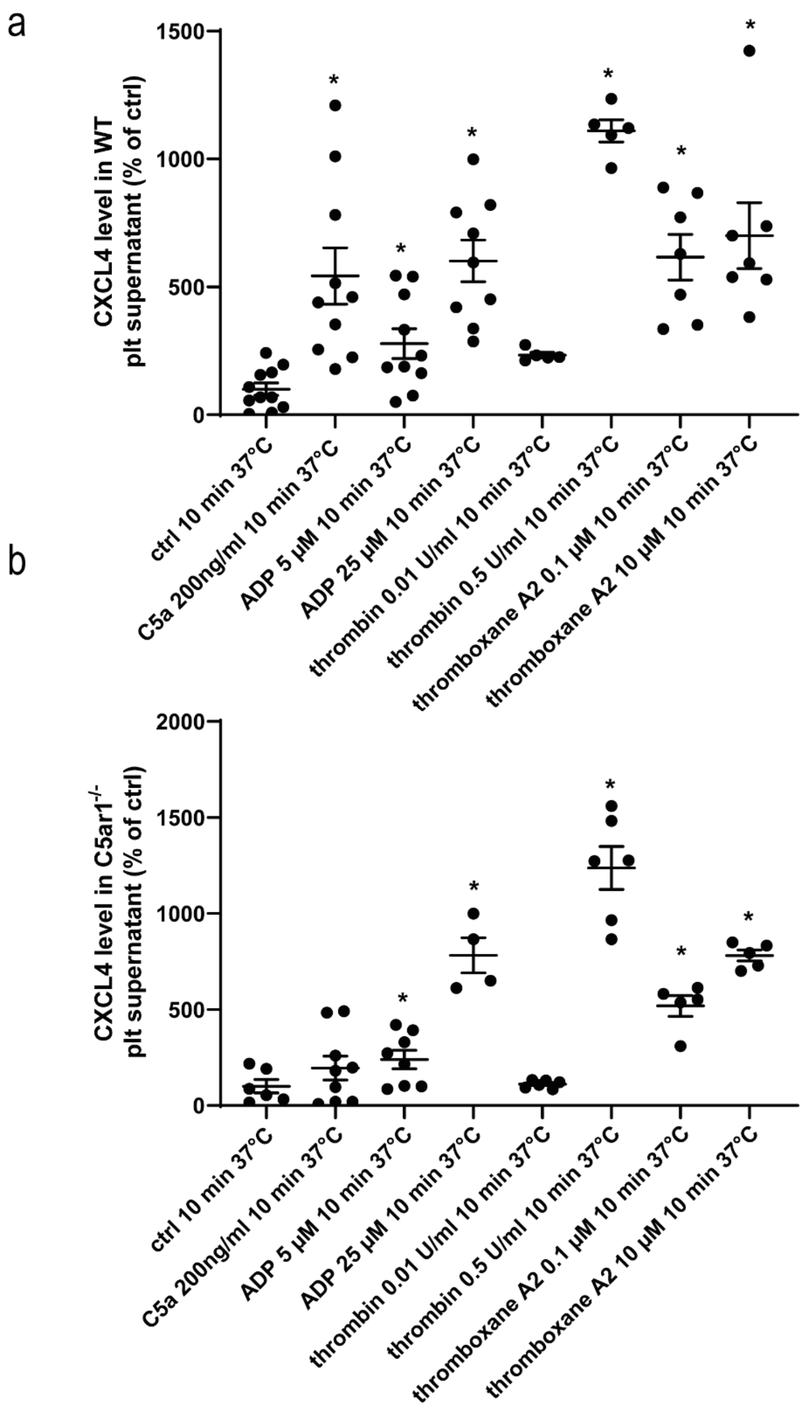

Supplementary figure 13: Characteristics of CXCL4 release from platelets upon stimulation with different agonists

(a) Washed murine WT platelets were stimulated with different agonists at different concentrations for 10 minutes at 37°C. The supernatant was analyzed for CXCL4 by ELISA. Data are shown as the mean±SEM (n=5-11) and are displayed as % of control. The CXCL4 protein level of vehicle-stimulated platelet supernatant represents 100%. *p<0.05. (b) Similarly, *C5ar1*$^{-/-}$ platelets were stimulated. No observable CXLC4 secretion can be detected in *C5ar1*$^{-/-}$ platelets upon C5a stimulation. Data are shown as the mean±SEM (n=4-10 independent experiments) of CXCL4 level measured by ELISA in platelet supernatant and are displayed as % of control. The CXCL4 protein level of vehicle-stimulated platelet supernatant represents 100%. One-way ANOVA with Bonferroni post-hoc correction in (a) – (b).

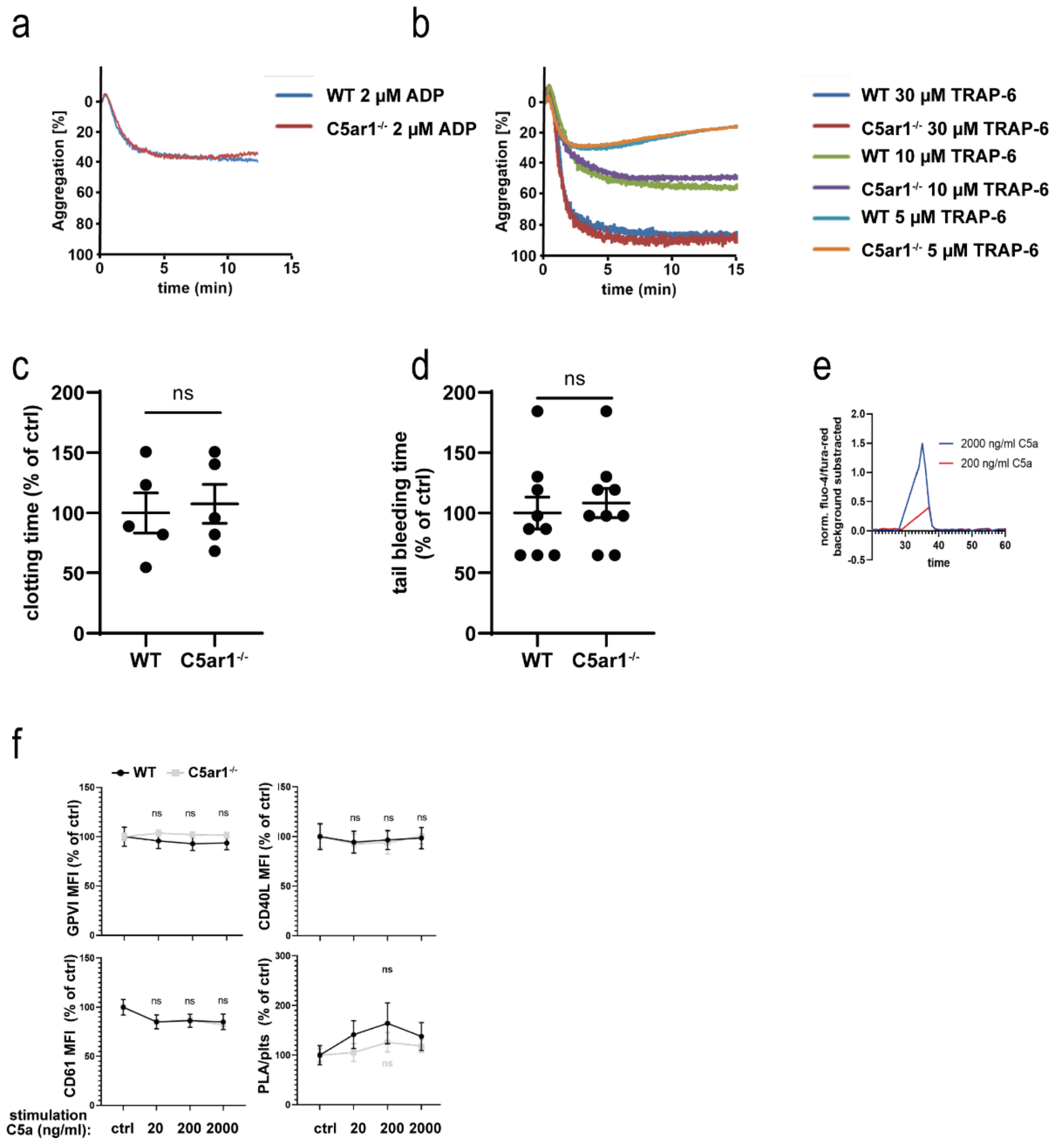

Supplementary figure 14: Influence of platelet C5aR1 expression on classical platelet functions such as aggregation, calcium signaling and thrombus formation or bleeding time.

(a) WT and *C5ar1*$^{-/-}$ platelets did not differ in aggregation in response to ADP as assessed by light transmission aggregometry. Shown is one representative experiment out of 3. (b) In response to TRAP-6 WT and *C5ar1*$^{-/-}$ platelets did not differ in their aggregation behavior.

(c) There was no difference in between time to vessel occlusion in ferric chloride induced vascular injury comparing WT and *C5ar1*$^{/-}$ mice. Data are shown as the mean±SEM (n=5 animals per group) and are displayed as % of control. The mean time to vessel occlusion in WT mice represents 100%. (d) Tail bleeding times of mice were assessed as described in the Methods section. There was no significant difference between WT and *C5ar1*$^{/-}$ mice. Data are shown as the mean±SEM (n=9 animals per group) and are displayed as % of control. The tail bleeding time in WT mice represents 100%. (e) Isolated platelets were washed and assessed by flow cytometry for a calcium signaling effect using fura-red and fluo-4 as described in the Methods section. C5a stimulation at a concentration of 200 ng/ml and 2000 ng/ml was able to induce a short calcium signal (baseline subtracted). Displayed is a representative graph of four independent experiments displayed as the ratio of fluo-4 over fura-red. Two (f) Citrated whole blood from WT and *C5ar1*$^{-/-}$, mice was stimulated using different agonists. C5a stimulation did not induce upregulation of activation parameters GPVI, CD61 and CD40L in WT and C5ar1-/- platelets. Also, platelet-leukocyte aggregate (PLA) formation was not increased. Data are

shown as the mean±SEM (n=4 independent experiments) and are displayed as % of control. The mean MFI of platelets in the vehicle-stimulated group represents 100%. (Two-way ANOVA with Bonferroni post-hoc correction in (f), Student's t-test in (c), (d).

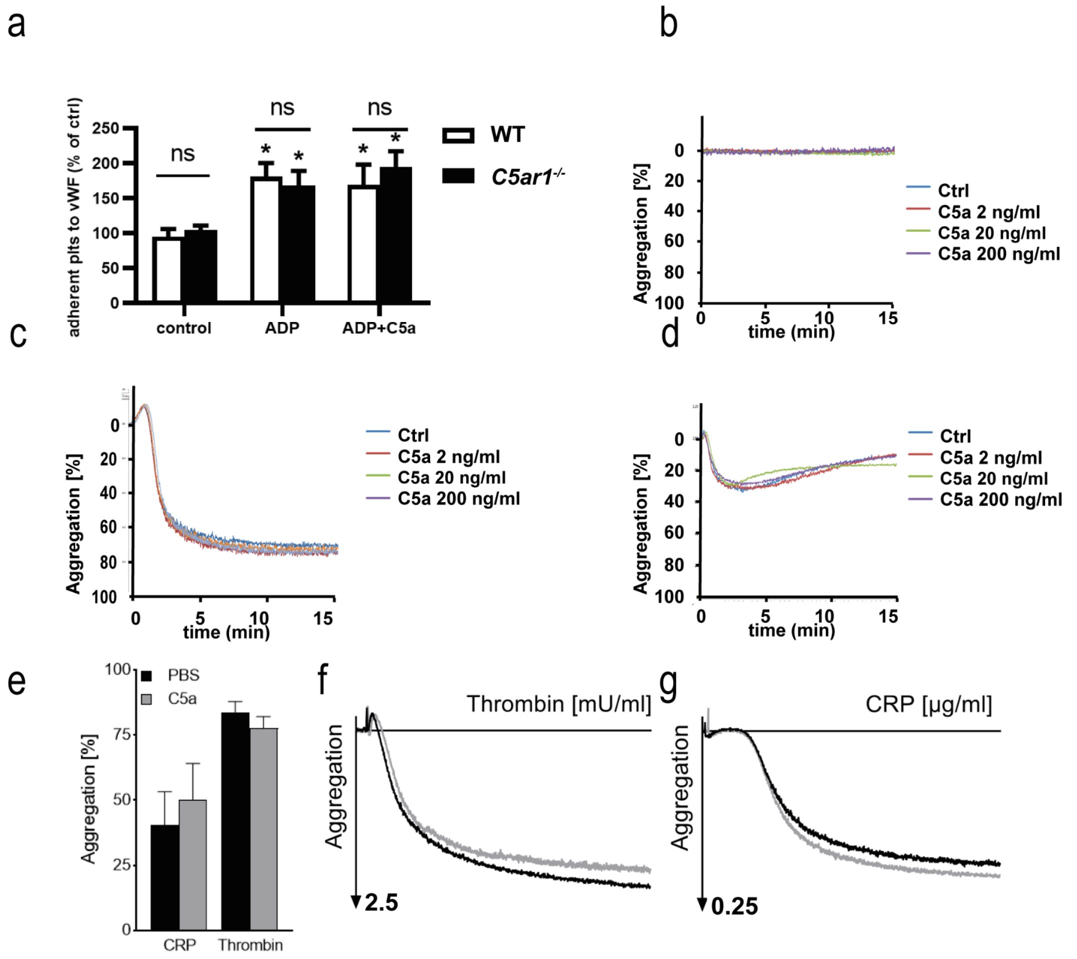

Supplementary figure 15: C5a does not alter platelet adhesion to fibrinogen or platelet aggregation

(a) In order to assess classical platelet functions, we performed a static adhesion assay. Isolated washed murine WT and *C5ar1*$^{-/-}$ platelets (1 x 10^6 / well) were stimulated with vehicle control, ADP (10 µM) or C5a (20ng/ml) and left to adhere to a fibrinogen coated 96 well plate for 1h at RT. Pictures were taken microscopically and a particle analysis was performed using ImageJ software. No significant differences in adhesion between WT and *C5ar1*$^{-/-}$ platelets could be detected. Data are shown as the mean±SEM (n=5 independent experiments) and as % of control. The number of adherent platelets in the WT group with vehicle control stimulation represent 100%. (b) Platelet aggregation was analyzed in murine WT platelets using conventional aggregometry. Platelet aggregation was not altered by stimulation with C5a alone. (c) Murine WT platelets were stimulated with 2 µM ADP and different dosages of C5a. No relevant differences in aggregation were detected. One representative experiment of 3 repeats is depicted. (d) Similarly, platelets were stimulated with 10 µM TRAP and different concentrations of C5a. (e) Freshly isolated WT platelets were stimulated with C5a for 10 minutes at 37°C and then, CRP or thrombin were added and aggregation was monitored. Data are shown as the mean±SEM (n=6 independent experiments). (b), (c), (d), (f) and (g) show representative aggregometer curves or 4 independent experiments. One-way ANOVA with Bonferroni post-hoc correction in (a), (e).

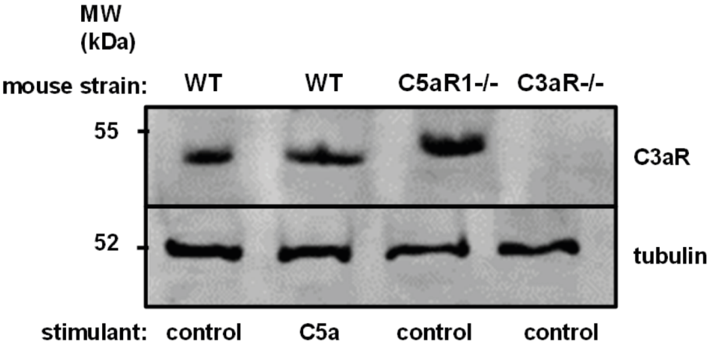

Supplementary figure 16: Impact of C5aR1-knockout on C3aR protein levels in platelets
The level of C3aR was assessed after C5a stimulation (20 ng/ml) or vehicle control stimulation in WT or *C5ar1*$^{-/-}$ platelet lysates. No significant differences were detected. Lysates from *C3ar*$^{-/-}$ mice served as a control. Displayed is a representative image from 4 independent experiments.

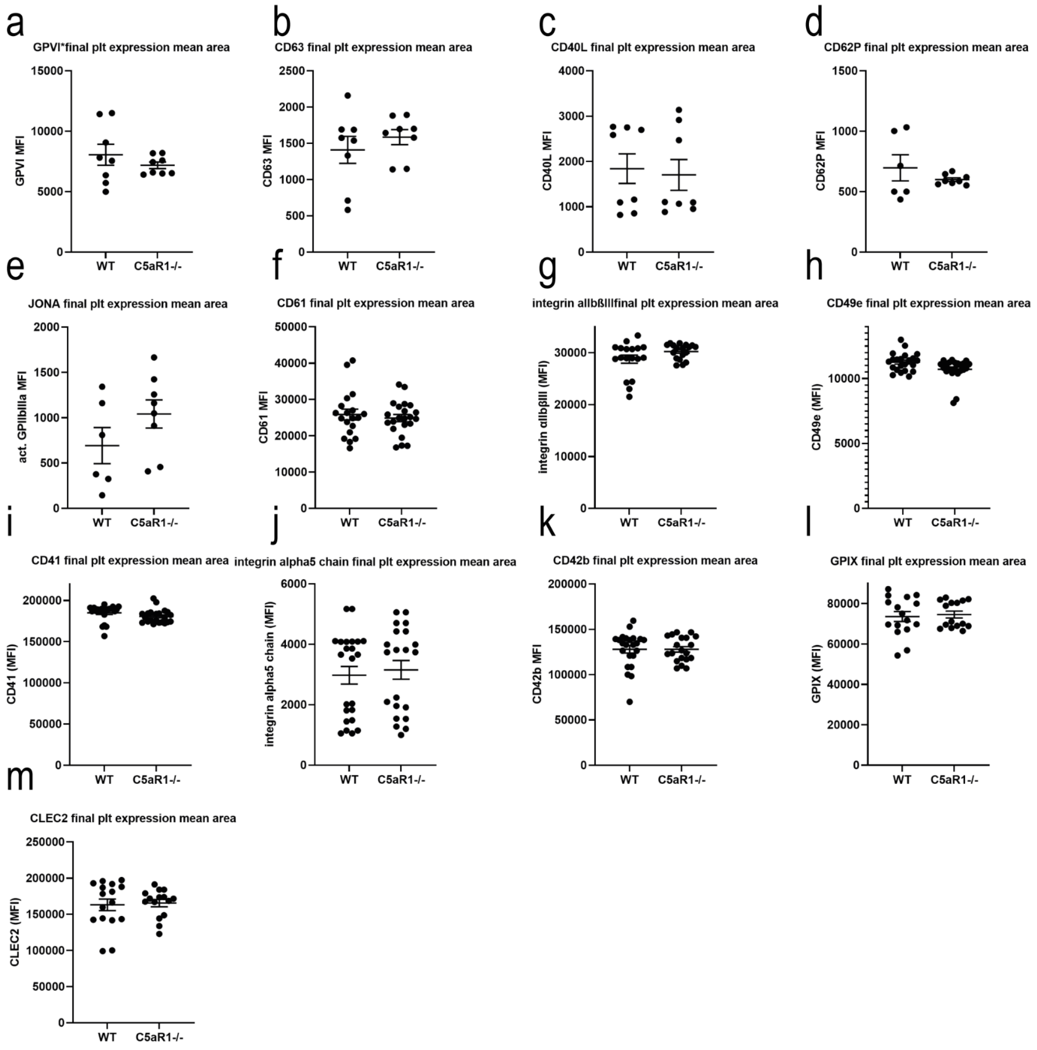

Supplementary figure 17: Platelet surface marker expression in WT vs. *C5ar1$^{-/-}$* platelets (a) Platelets were stained in whole citrated murine blood and adhesion receptor expression was quantified in WT vs. *C5ar1$^{-/-}$* mice. No significant differences were detected in GPVI expression (a), CD63 expression (b), CD40L expression (c), CD62P expression (d), activated GPIIbIIIa expression (e), CD61 expression (f), basal GPIIbIIIa (g), CD493 expression (h), CD41 expression (i), integrin α 5 chain expression (j), CD42b expression (k), GPIX expression (l), Clec2 expression (m). All data are shown as the mean±SEM (n=6-23 independent measurements) and are displayed as MFI. Student's t-test in (a) – (m).

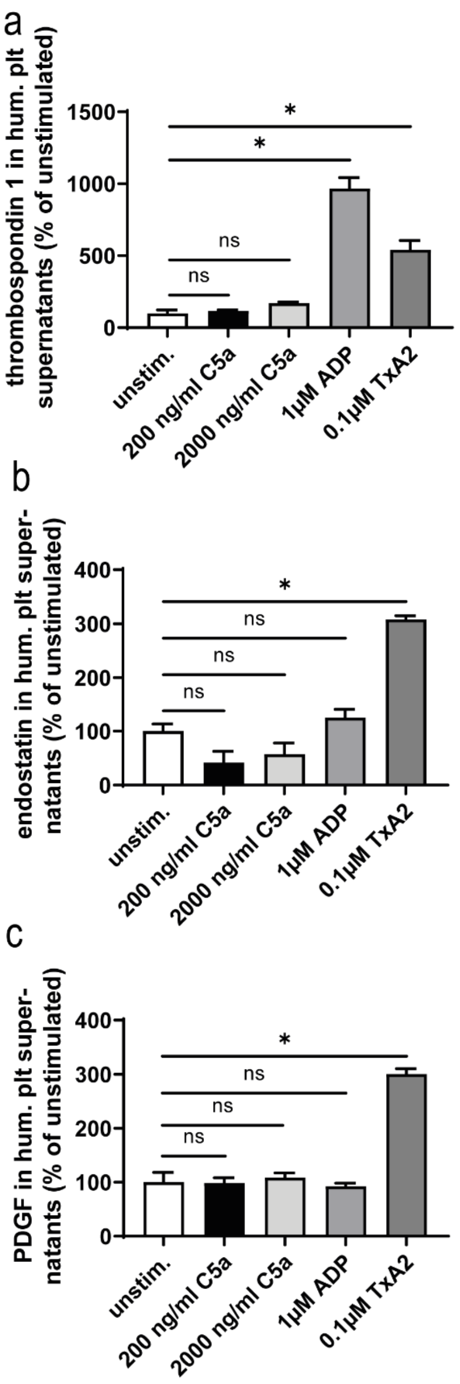

Supplementary figure 18: Release of various angiogenic compounds from platelets upon stimulation

(a) Washed human platelets were stimulated with different agonists at different concentrations for 10 minutes at 37°C. The supernatant was analyzed for thrombospondin 1, endostatin and PDGF by ELISA. (a-c) TxA2 induced secretion of all factors. A low dose of ADP only induced thrombospondin 1 secretion, C5a did not induce significant secretion of any of these factors compared to ADP or TxA2. All data are shown as the mean±SEM (n=4 independent experiments) and are displayed as % of control. $^*p<0.05$. One-way ANOVA with Bonferroni post-hoc correction in (a) – (c).

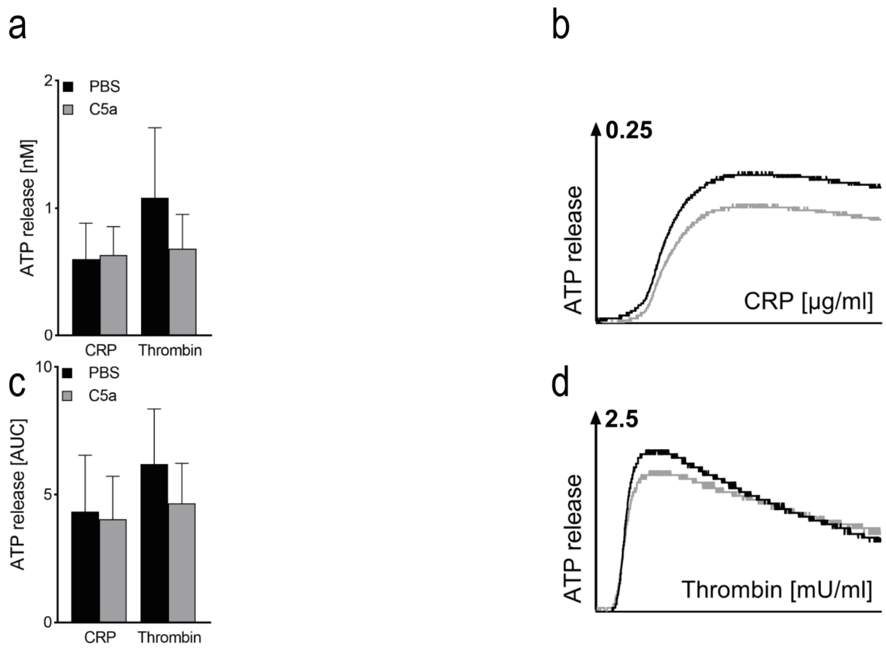

Supplementary figure 19: Impact of C5a on agonist-induced ATP release from platelets (a) Washed murine WT platelets were stimulated with C5a (20 ng/ml) for 10 minutes at room temperature. Then, ATP release upon stimulation with CRP and thrombin was assessed by lumi-aggregometry. No significant impact of C5a on agonist-induced ATP release was detectable. Data are shown as the mean±SEM (n=5 independent experiments) and are displayed as nM ATP. (b) Representative ATP release curve after CRP stimulation. (c) The same experiment as in (a) was performed with C5a at 200 ng/ml. (b) Representative ATP release curve after thrombin stimulation. One-way ANOVA with Bonferroni post-hoc correction in (a), (c).

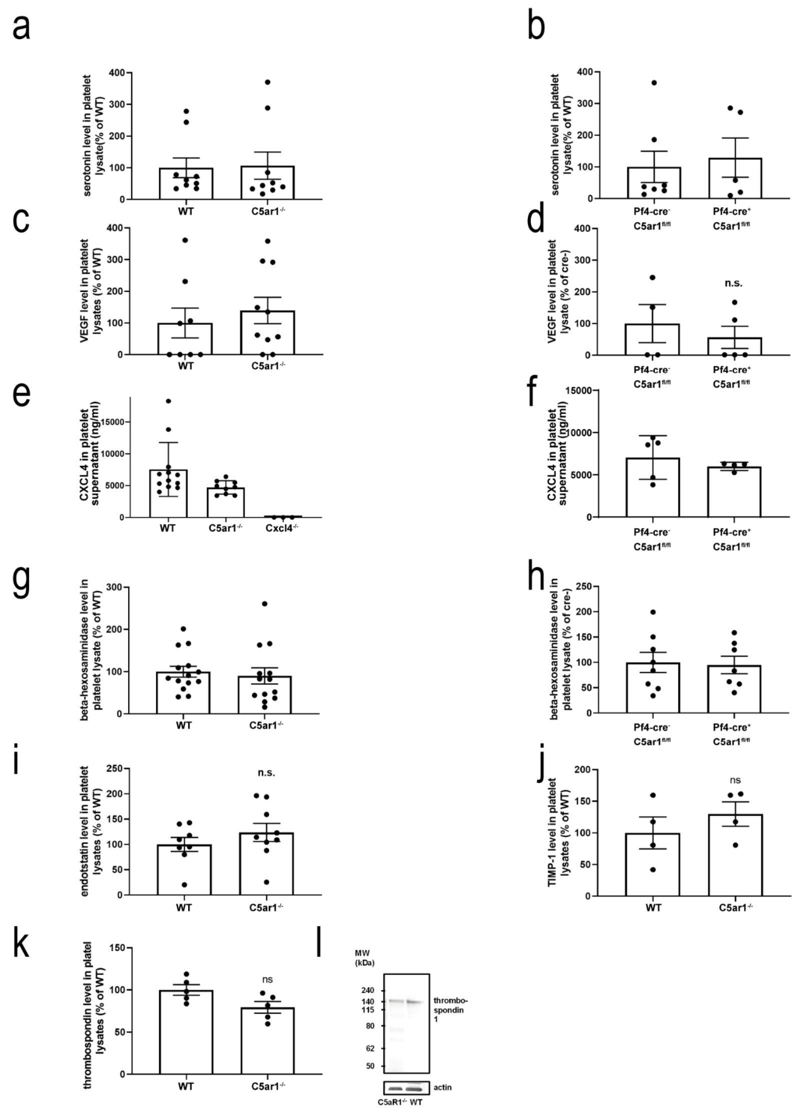

Supplementary figure 20: Granule content characteristics of WT vs. *C5ar1*$^{-/-}$ and *Pf4-cre*$^{+}$*C5ar1*$^{fl/fl}$ vs. *Pf4-cre*$^{-}$*C5ar1*$^{fl/fl}$ platelets

Freshly isolated washed murine platelets were isolated and lysed. In none of the experiments, significant differences between WT versus *C5ar1*$^{-/-}$ platelets or *Pf4-cre*$^{+}$*C5ar1*$^{fl/fl}$ vs. *Pf4-cre*$^{-}$*C5ar1*$^{fl/fl}$ platelets were detectable. (a) and (b) Serotonin was measured by ELISA. (c) and (d) VEGF was measured by ELISA. (e) and (f) CXCL4 was measured by ELISA. Platelets isolated from CXLC4$^{-/-}$ mice were used as a control. (g) and (h) Beta-hexosaminidase was measured by ELISA. (i) and (j) Endostatin was measured by ELISA. (k) and (l) Thrombospondin was measured by Western blotting. 5 blots were quantified relative to actin as loading control. No significant differences could be detected between WT and *C5ar1*$^{-/-}$ platelets. All data are shown as the mean±SEM (n=1-14 independent experiments) and are displayed as % of control. The protein level of WT platelet lysates or *Pf4-cre*$^{-}$*C5ar1*$^{fl/fl}$ platelet lysates measured by ELISA represents 100% in each graph. Student’s t-test in (a) – (d), (f) – (k). One-way ANOVA with Bonferroni post-hoc correction in (e).

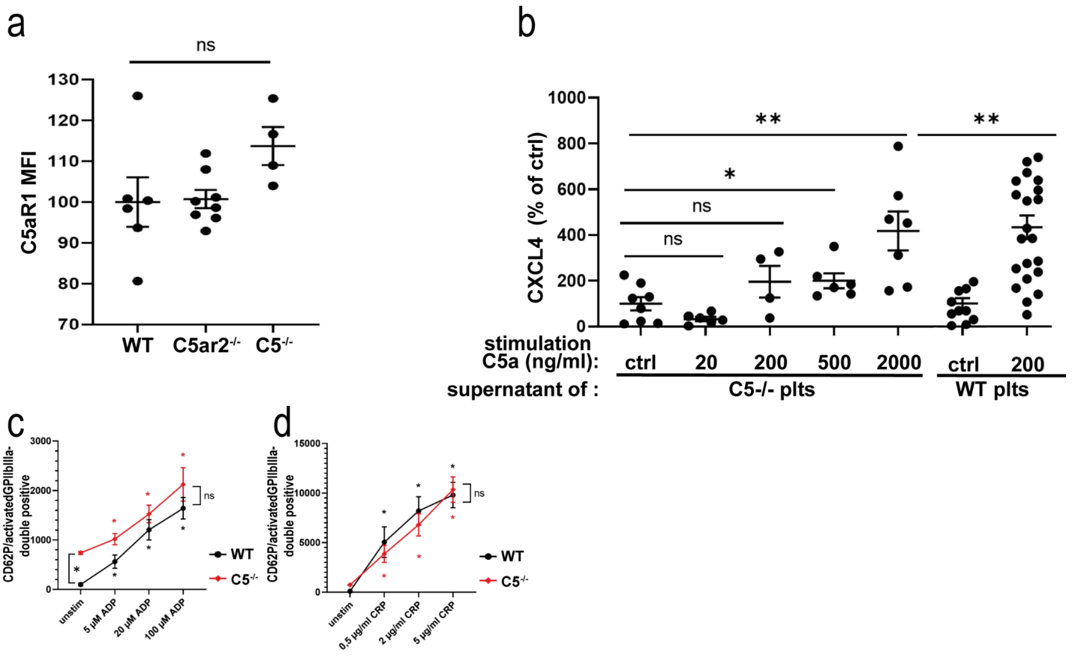

Supplementary figure 21: Characterization of *C5*$^{-/-}$ platelets

(a) In whole citrated murine blood, C5aR1 expression was assessed on $CD41^{+}$ platelets by flow cytometry. *C5*$^{-/-}$ platelets displayed a slightly higher C5aR1 expression, which was not statistically significant. Data are shown as the mean±SEM (n=5-8 independent measurements) and as % of control. The MFI measurement of C5aR1 on platelets from WT mice represents 100%. (b) Washed murine *C5*$^{-/-}$ platelets or WT platelets were stimulated with different concentrations of C5a for 10 minutes at 37°C. The supernatant was analyzed for CXCL4 by ELISA. Significant CXCL4 secretion was only detectable at higher C5a concentrations (500 and 2000 ng/ml) and was overall lower than in the WT control group. Data are shown as the mean±SEM (n=4-21 independent measurements) and as % of control. The CXCL4 level in vehicle control stimulated C5-/- supernatant represents 100%. (c) Citrated whole blood from WT and C5-/- mice was stimulated using different concentration of ADP (5, 20, 100 µM) or CRP (0.5, 2, 5 µg/ml) and vehicle control and assessed for platelet activation markers using flow cytometry. For the gating strategy please refer to the Methods section. Activated platelets were defined as CD62P/activated GPIIbIIIa-double positive and are expressed as % gated. C5-/- platelets display a slightly higher basal activation level. However, after activation with ADP (c) and CRP (d), there is no significant difference in comparison with WT platelets. In (c) and (d) data are shown as the mean±SEM (n=4 independent experiments) and are displayed as % of control. The mean fluorescence intensity (MFI) of platelets in the vehicle-stimulated group represents 100% in all graphs. *$p<0.05$. One-way ANOVA with Bonferroni post-hoc correction in (a), (b). Two-way ANOVA with Bonferroni post-hoc correction in (c), (d).

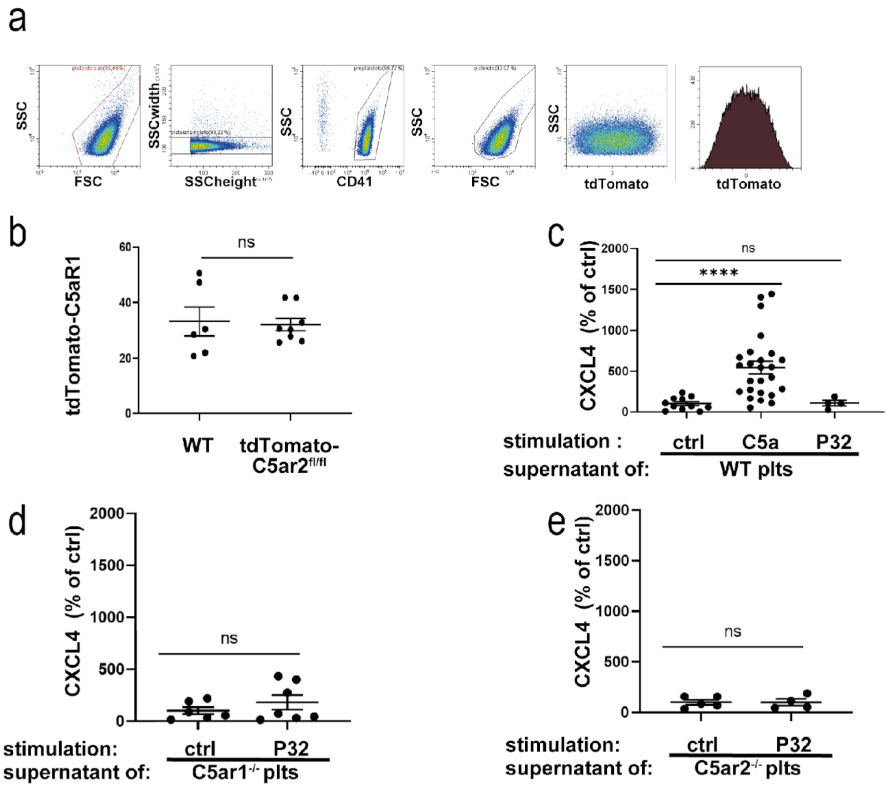

Supplementary figure 22: C5aR2 does not alter platelet C5aR1-dependent CXCL4 secretion

(a) In order to assess C5aR2 expression on platelets, tdTomato-C5aR2 reporter mice were analyzed by flow cytometry. Shown is the gating strategy to measure single platelets. b) No difference was observed in the tdTomato signal in platelets from tdTomato-C5ar2 and WT mice. Data are shown as the mean±SEM (n=6-8 independent measurements involving different animals) and are displayed as median fluorescence intensity (MFI) of platelets measured by flow cytometry. (c) Washed murine WT platelets were stimulated with C5a (200 ng/ml) and the C5aR2-agonist p32 (1 µM) for 10 minutes at 37°C. The supernatant was analyzed for CXCL4 by ELISA. P32 did not induce significant CXCL4 secretion. Data are shown as the mean±SEM (n=5-25 independent experiments) and are % of control. The CXCL4 level in vehicle control-stimulated WT supernatant represents 100%. *p<0.05. (d) Similarly, *C5ar1*$^{-/-}$ or (e) *C5ar2*$^{-/-}$ platelets were stimulated and the supernatant was probed for CXCL4. In *C5ar1*$^{-/-}$ mice, p32 did not induce CXLC4 secretion. For both (d) and (e) data are shown as the mean±SEM (n=5-8 independent experiments) of the level of CXCL4 measured by ELISA in platelet supernatant and are % of control. The CXCL4 level in the vehicle control-stimulated group represents 100%. One-way ANOVA with Bonferroni post-hoc correction in (c) and Student's t-test in (b), (d), (e).

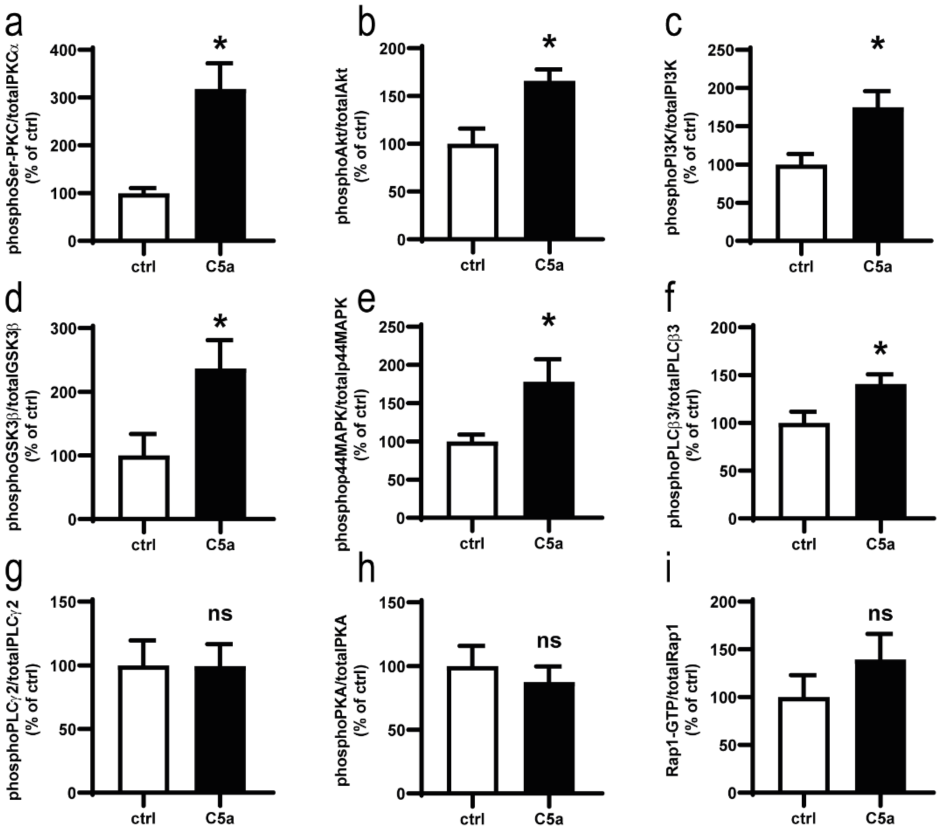

Supplementary figure 23: C5aR1 signaling in platelets

Lysates of WT platelets were generated after vehicle control or C5a stimulation and samples were probed at equal protein concentrations for phospho-proteins as well as non-phosphorylated controls. (a) – (h) Phosphorylation was quantified as phosphorylated intensity over total protein intensity. Data are shown as the mean±SEM (n=4 samples measured and quantified) and are displayed as % of control. The mean intensity of phosphorylated over total protein of platelet lysate from platelets stimulated with vehicle-control represents 100% in all graphs. (i) Rap1 activation was quantified as Rap1-GTP over total Rap1. Data are shown as the mean±SEM (n=4 samples measured and quantified) and are displayed as % of control. The mean intensity of Rap1-GTP over total Rap1 of platelet lysate from platelets stimulated with vehicle-control represents 100%. *$p<0.05$. Student's t-test in (a) – (i).

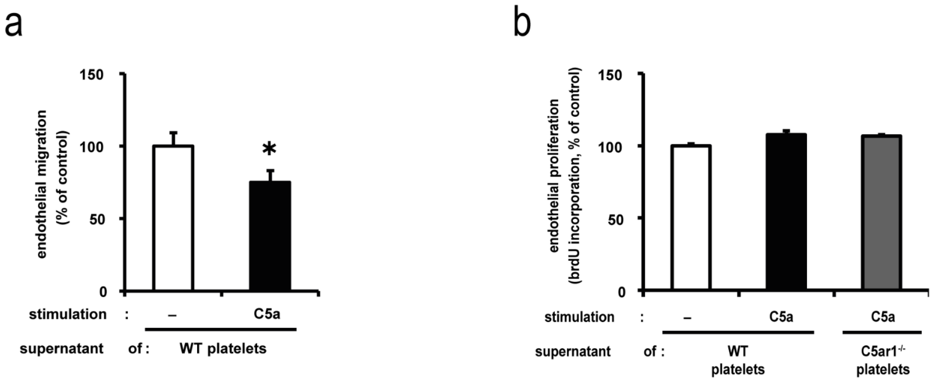

Supplementary figure 24: C5a-conditioned platelet supernatant has no impact on endothelial proliferation but inhibits endothelial migration

(a) Migration after endothelial injury was significantly inhibited by C5a-stimulated platelet supernatant compared with supernatant from platelets treated with vehicle control. Data are presented as the mean±SEM (n=4 independent experiments) and are shown as % of control. The total area repopulated with cells in the group treated with vehicle-stimulated platelet supernatant represents 100%. *p<0.05. (b) The effect of C5a-conditioned platelet supernatant on endothelial proliferation of MHEC-5T cells was assessed using a BrdU-incorporation ELISA after 18 h. Data are displayed as the mean±SEM (n=5 independent experiments). The BrdU-incorporation measurement of cells stimulated with vehicle control represents 100%. Student's t-test in (a), one-way ANOVA with Bonferroni's post hoc test in (b).

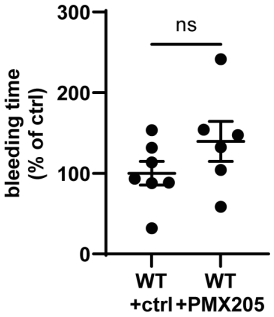

Supplementary figure 25: The C5aR1 antagonist PMX205 has no effect on bleeding time
Tail bleeding times of mice were assessed as described in the Methods section. There was no significant difference between WT mice treated with the C5aR1-antagonist PMX205 or mice treated with a control peptide regarding time to complete bleeding cessation. Data are shown as the mean±SEM (n=7 animals per group) and are displayed as % of control. The tail bleeding time in WT mice treated with control peptide represents 100%. Two-sided Student's t-test.

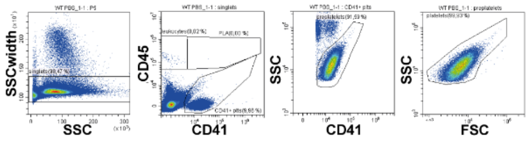

Supplementary figure 26: Gating for platelets in citrated full blood was performed by excluding doublets using SSCwidth versus SSC characteristics. After that, CD41 and CD45 were used to identify platelets, leukocytes and platelet-leukocyte aggregates (PLA). Furthermore, the platelet population was further differentiated from debris using FSC and SSC at a logarithmic scale.

Supplementary Note 1: Antibody List

R&D:
rat anti-mouse anti-CXCL4 MAB, IgG2b, Clone 140910, #MAB595-100
anti-CXCL4 antibody (rat IgG2b, MAB 595, R&D) (AF595)

BioLegend:
PE anti-mouse CD88 (C5aR) Antibody**,** clone 20/70, BioLegend, # 135805;
APC anti-mouse CD88 (C5aR) Antibody, clone 20/70, Biolegend, #135808;
Brilliant Violet 605™ anti-mouse/human CD11b Antibody, clone M1/70, BioLegend, #101257;
Brilliant Violet 785™ anti-mouse F4/80 Antibody, clone BM8, BioLegend, #123141;
PE/Dazzle™ 594 anti-mouse/rat CD61 Antibody, clone 2C9.G2, BioLegend, #104321;
PE/Cyanine7 anti-mouse CD154 Antibody, clone MR1, BioLegend, #106512;
Brilliant Violet 605™ anti-mouse CD45 Antibody, clone 30-F11, BioLegend, #103139;
Pacific Blue™ anti-mouse CD41 Antibody, clone MWReg30, BioLegend, #133932;
PE anti-mouse CD49e Antibody, clone 5H10-27(MFR5), BioLegend, #103805;
PE anti-mouse CLEC-2 (CLEC1B) Antibody, clone 17D9/CLEC-2, BioLegend, #146103;
APC anti-mouse CD14 Antibody, clone Sa14-2, BioLegend, #123312;
APC anti-mouse CD31 Antibody, clone MEC13.3, BioLegend, #102510;
rat anti-mouse CD88 (C5aR) Antibody, BioLegend #135815.

Emfret Analytics:
PE- and FITC- labeled antibodies, Clones JON/A / Wug.E9, Emfret Analytics, #D200;
FITC-labeled Rat Anti-Mouse GPVI Monoclonal Antibody, Clone JAQ1, Emfret Analytics, #M011-1;
Integrin alphaIIbbeta3 (GPIIb/IIIa, CD41/CD61), clone Leo.F2, Emfret Analytics, #M025-1;
GPIbalpha (CD42b), clone Xia.G5, Emfret Analytics, #M040-2;
Integrin alpha5 chain (CD49e), clone Tap.A12, Emfret Analytics, #M080-1;
GPIX (CD42a), clone Xia.B4, Emfret Analytics, #M051-1;
Dylight 488 conjugated CD41 antibody, Emfret Analytics, #X488;

eBioscience:
CD45 Mouse anti-Human, PE-Cyanine5.5, Clone: HI30, eBioscience, #350459-42;
CD144 (VE-cadherin) Monoclonal Antibody (eBioBV13 (BV13)), Alexa Fluor 488, eBioscience, #53-1441-82.

Invitrogen:
Isolectin GS-IB4 Alexa Fluor 594. - Isolectin GS-IB_4 From *Griffonia simplicifolia*, Alexa Fluor™ 594 Conjugate,
Invitrogen, # I21413;
donkey anti-goat Alexa Fluor 488 antibody, - Donkey anti-Goat IgG (H+L) Cross-Adsorbed Secondary Antibody, Alexa Fluor 488, Invitrogen, # A-11055;
goat anti-rat Alexa Fluor 568 antibody - Goat anti-Rat IgG (H+L) Cross-Adsorbed Secondary Antibody, Alexa Fluor 568, Invitrogen, #A11077;
goat anti-rabbit Alexa Fluor 488 antibody, # A-11008

Merck:
anti-NG2 Chondroitin Sulfate Proteoglycan Antibody, Merck, # AB5320;

Abcam:
Goat Anti-Rat IgG HRP-conjugated Antibody, (Abcam, ab205718, 1:2000)
rat anti-mouse anti-C3 antibody, clone 11H9, Abcam, ab11862, 1:20)
phospho PKA (PKA α/β/γ catalytic subunit phospho T197), Abcam, #ab75991, 1:3000),
total PKA (α/β catalytic subunits, Abcam, #ab216572, 1:500);
anti-β-actin (Abcam, #8226, 1:1000).
rat anti-mouse anti-C3 antibody (clone 11H9, ab11862, Abcam, Milton, UK, 1:20)
donkey anti-rat preadsorbed IgG Alexa Fluor 568 (ab175475, Abcam, 1:500)
donkey anti-mouse preadsorbed IgG Alexa Fluor 488 (ab150109, Abcam, 1:500)
goat anti-rabbit IgG coupled with biotin (ab6720, Abcam, 1:5000)

Goat Anti-Rabbit IgG H&L (HRP) (ab205718)
Goat Anti-Mouse IgG H&L (HRP) (ab205719)

Dianova:
Donkey F(ab')2 anti-rat IgG (H+L)-Alexa Fluor 647, Dianova, #712-606-153;
Donkey anti-Rabbit IgG (H+L)-Cy3, Dianova, # 711-165-152;
Donkey F(ab')2 anti-Rabbit IgG (H+L)-Alexa Fluor 647, Dianova, # 711-606-152;

LI-COR:
IRDye® 800CW Goat anti-Rat IgG Secondary Antibody, Li-COR, #926-32219;

Novus Biologicals:
anti-mouse CD63-PECy5.5, clone NKI/C3, Novus Biologicals, # NBP2-34694PECY55;

Santa Cruz:
α-actin antibody, clone1A4, Santa Cruz, # sc-32251;
goat anti-mouse C5aR1 antibody cone P14, CD88, Santa Cruz, # sc-3124
mouse monoclonal P-selectin antibody, clone CTB201, Santa Cruz, #sc-8419;
C3aR Antikörper, clone D-12, Santa Cruz, # sc-133172;

Cloud Clone Corporation:
Polyclonal Antibody to Platelet Factor 4 (PF4), Cloud Clone Corporation, # PAA172Mu01;

Sigma Aldrich:
Monoclonal anti-α-Tubulin-antibody, clone B-5-1-2, Sigma Aldrich, #T5168;

Hycult Biotech:
rat anti-CD88 antibody, clone 10/92, Hycult, # HM1077-100UG;

BD Biosciences:
rat anti-mouse anti-CD102 Clone 3C4 - Purified Rat Anti-Mouse CD102, Clone 3C4(mIC2/4), BD Biosciences, #553326;
purified rat anti-mouse CD62P - Purified Rat Anti-Mouse CD62P, Clone RB40.34, BD Biosciences, #550289;

Proteintech:
thrombospondin 1 (rabbit polyclonal Anti-Thrombospondin-1 Antibody, Proteintech, Chicago, Illinois, USA, #1 18304-1-AP;

Thermo Fisher Scientific:
Donkey anti-Rat IgG (H+L) Highly Cross-Adsorbed Secondary Antibody, Biotin, Invitrogen, # A18749;
Donkey anti-Rabbit IgG (H+L) Highly Cross-Adsorbed Secondary Antibody, Alexa Fluor 647, Invitrogen, # A-31573;
Goat anti-Rat IgG (H+L) Cross-Adsorbed Secondary Antibody, Alexa Fluor 488, Invitrogen, # A-11006;

Cell Signaling Technology:

phospho PLCγ2 (Tyr1217, Cell Signaling Technology, #3871S, 1:1000), total PLCγ2 (Cell Signaling Technology, Danvers, USA, #3872S, 1:1000);
phospho Akt (Ser473, Cell Signaling Technology, #9271S, 1:1000),
total Akt (Rabbit monoclonal Akt pan C67E7, Cell Signaling Technology, # 4691S, 1:1000);
phospho PI3K (Phospho PI3 Kinase p85 Tyr458/p55 Tyr199, Cell Signaling Technology, #4228S, 1:1000),
total PI3K (rabbit monoclonal PI3 Kinase p85 19H8, Cell Signaling Technology, #4257S, 1:1000);
phospho GSK 3β (Ser9, Cell Signaling Technology, #9336S, 1:1000),
total GSK 3β (rabbit monoclonal anti GSK 3β 27C10, Cell Signaling Technology, #9315S, 1:1000);
phospho p44/42 MAPK (Erk1/2 Thr202/Tyr204, Cell Signaling Technology, #9101S, 1:1000),
total p44/42 MAPK (Erk1/2, Cell Signaling Technology, #9102S, 1:1000);
phospho PLC β 3 (Ser537, Cell Signaling Technology, #29021S, 1:1000),
total PLC β 3 (rabbit monoclonal PLCβ3 D9D6S, Cell Signaling Technology, #14247, 1:1000);
phospho PKC (Ser) substrate (Cell Signaling Technology, #2261S, 1:1000),
total PKCα (Cell Signaling Technology, #2056S, 1:1000).

<u>ELISAs:</u>
mouse PF4/CXCL4 Quantikine ELISA Kit (R&D) , a membrane-based antibody array (Proteome Profiler Mouse Angiogenesis Array Kit, ARY015, R&D),
a Serotonin ELISA kit (BA E-8900, LDN, Nordhorn, Germany),
a Hexosaminidase B B (HEXb) ELISA Kit (SEA637Mu, Cloud Clone Corporation),
a Mouse VEGF ELISA Kit (ab209882, Abcam),
a Thrombospondin 1 ELISA Kit (THBS1, ABIN6574175, Antibodies-Online, Aachen, Germany),
an Endostatin COL18A1/ES ELISA kit (Mouse collagen type XVIII α 1 Endostatin ELISA Kit, MBS701673, MyBioSource.com, San Diego, USA)
and a TIMP-1 ELISA Kit (Mouse TIMP-1 Quantikine ELISA, MTM100, R&D).
human CXCL4/PF4 Quantikine ELISA kit (MCX400, R&D),
a human PDGF BB ELISA kit (ab100624, Abcam)
