# Supplementary material for: The C5a/C5a receptor 1 axis controls tissue neovascularization through CXCL4 release from platelets
Source: Nat Commun. 2021 Jun 7;12:3352. doi: 10.1038/s41467-021-23499-w (PMC8185003; doi:10.1038/s41467-021-23499-w)
Supplement: Supplementary file 3 — Description of Additional Supplementary Files [file 41467_2021_23499_MOESM3_ESM.pdf]

Description of Additional Supplementary Files

File Name: Supplementary Data 1
Description: List of primers used for PCR

File Name: Supplementary Movies 1a-d
Description: **C5aR1 deficiency on platelets induces increased collateral artery formation**
Platelet-specific C5aR1-deficient mice and control were subjected to hindlimb ischemia. To visualize collateral arteries, mice were perfused with a contrast agent at d9 after induction of ischemia and subjected to microCT analysis as described in the Methods section. 3D reconstructions of arteries from ischemic hindlimbs showed collateral artery formation, which was reconstructed as a 3D model. The videos depict representative reconstructions from (a) nonischemic hindlimbs of *Pf4-cre*$^{-}$ *C5ar1*$^{fl/fl}$ mice; (b) ischemic hindlimbs of *Pf4-cre*$^{-}$ *C5ar1*$^{fl/fl}$ mice; (c) nonischemic hindlimbs of *Pf4-cre*$^{+}$*C5ar1*$^{fl/fl}$ mice; (d) ischemic hindlimbs of *Pf4-cre*$^{+}$*C5ar1*$^{fl/fl}$ mice.
