# Supplementary material for: The C5a/C5a receptor 1 axis controls tissue neovascularization through CXCL4 release from platelets
Source: Nat Commun. 2021 Jun 7;12:3352. doi: 10.1038/s41467-021-23499-w (PMC8185003; doi:10.1038/s41467-021-23499-w)
Supplement: Supplementary file 9 — Reporting Summary [file 41467_2021_23499_MOESM9_ESM.pdf]

nature research

Corresponding author(s): Harald F. Langer

Last updated by author(s): March 16 2021

Reporting Summary

Nature Research wishes to improve the reproducibility of the work that we publish. This form provides structure for consistency and transparency in reporting. For further information on Nature Research policies, see our Editorial Policies and the Editorial Policy Checklist.

Statistics

For all statistical analyses, confirm that the following items are present in the figure legend, table legend, main text, or Methods section.

n/a	Confirmed	
☐	☒	The exact sample size (*n*) for each experimental group/condition, given as a discrete number and unit of measurement
☐	☒	A statement on whether measurements were taken from distinct samples or whether the same sample was measured repeatedly
☐	☒	The statistical test(s) used AND whether they are one- or two-sided<br>*Only common tests should be described solely by name; describe more complex techniques in the Methods section.*
☒	☐	A description of all covariates tested
☐	☒	A description of any assumptions or corrections, such as tests of normality and adjustment for multiple comparisons
☐	☒	A full description of the statistical parameters including central tendency (e.g. means) or other basic estimates (e.g. regression coefficient) AND variation (e.g. standard deviation) or associated estimates of uncertainty (e.g. confidence intervals)
☐	☒	For null hypothesis testing, the test statistic (e.g. *F*, *t*, *r*) with confidence intervals, effect sizes, degrees of freedom and *P* value noted<br>*Give P values as exact values whenever suitable.*
☒	☐	For Bayesian analysis, information on the choice of priors and Markov chain Monte Carlo settings
☒	☐	For hierarchical and complex designs, identification of the appropriate level for tests and full reporting of outcomes
☒	☐	Estimates of effect sizes (e.g. Cohen's *d*, Pearson's *r*), indicating how they were calculated

Our web collection on statistics for biologists contains articles on many of the points above.

Software and code

Policy information about availability of computer code

Data collection	Moor LDI image acquisition software (Moor Instruments, Ver. 5.3), Zen acquisition software 2.3 (Zeiss, Ver. 2012), LightCycler software (v.1.5, Roche), Nikon NIS elements BR software (v.3.2, Nikon), Axiovision software (v.3.2, Zeiss), CytExpert software (c.2.4), BZ-Analyzer X-800 software Ver 1.1.2 (Keyence)
Data analysis	Moor LDI image processing software (Moor Instruments, Ver. 5.3), Image Pro Plus Software (Ver. 7.0, Media Cybernetics), FloJo software (v.10, Tree Star), AngioTool software (National Cancer Center), ImageJ (current version, NIH), aggrolink8 software (ChronoLog), CytExpert software (c.2.4), ImageJ Vers. 1.5.3g; AngioTool ver. 0.5, GraphPad Prism (Ver. 9.0.1)

For manuscripts utilizing custom algorithms or software that are central to the research but not yet described in published literature, software must be made available to editors and reviewers. We strongly encourage code deposition in a community repository (e.g. GitHub). See the Nature Research guidelines for submitting code & software for further information.

Data

Policy information about availability of data

All manuscripts must include a data availability statement. This statement should provide the following information, where applicable:

- Accession codes, unique identifiers, or web links for publicly available datasets
- A list of figures that have associated raw data
- A description of any restrictions on data availability

Provide your data availability statement here.

Field-specific reporting

Please select the one below that is the best fit for your research. If you are not sure, read the appropriate sections before making your selection.

☒ Life sciences ☐ Behavioural & social sciences ☐ Ecological, evolutionary & environmental sciences

For a reference copy of the document with all sections, see nature.com/documents/nr-reporting-summary-flat.pdf

Life sciences study design

All studies must disclose on these points even when the disclosure is negative.

Sample size

Sample sizes of the hindlimb ischemia experiments were calculated with G*Power (freeware, Faul 2007). For all other experiments, no statistical methods were used to predetermine sample size. Where appropriate, all experiments were performed at least three times. Multiple animals and/or biological samples were measured in all assays, as is consistent with these types of studies.

Data exclusions

No data were excluded from analysis.

Replication

All experiments were performed as technical or biological replicates as appropriate for the experimental design and setup. The n-number is stated in the figure legends. All data are reported.

Randomization

No randomization occured. Due to small n-numbers randomization was not considered paramount for the overall quality of data. However, much care was given to comparability.

Blinding

During data collection and analysis, blinding to group allocations was performed at every instance for all experiments. During performance of experiments, blinding was not possible to ensure experimental accuracy and reduce potential sources of experementator error.
Specifically, animal experiments with heterogenic mouse litters (knockout-animals and littermate controls) were performed in a blinded fashion.
Qualitative readout of microCT images was performed in an experimentor-blinded fashion. Image analyses were performed in an experimentor-blinded fashion.

Reporting for specific materials, systems and methods

We require information from authors about some types of materials, experimental systems and methods used in many studies. Here, indicate whether each material, system or method listed is relevant to your study. If you are not sure if a list item applies to your research, read the appropriate section before selecting a response.

Materials & experimental systems

n/a	Involved in the study	
☐	☒	Antibodies
☐	☒	Eukaryotic cell lines
☒	☐	Palaeontology and archaeology
☐	☒	Animals and other organisms
☐	☒	Human research participants
☒	☐	Clinical data
☒	☐	Dual use research of concern

Methods

n/a	Involved in the study	
☒	☐	ChIP-seq
☐	☒	Flow cytometry
☒	☐	MRI-based neuroimaging

Antibodies

Antibodies used

R&D:
rat anti-mouse anti-CXCL4 MAB, IgG2b, Clone 140910, #MAB595-100
anti-CXCL4 antibody (rat IgG2b, MAB 595, R&D) (AF595)

BioLegend:
PE anti-mouse CD88 (C5aR) Antibody, clone 20/70, BioLegend, # 135805;
APC anti-mouse CD88 (C5aR) Antibody, clone 20/70, Biolegend, #135808;
Brilliant Violet 605™ anti-mouse/human CD11b Antibody, clone M1/70, BioLegend, #101257;
Brilliant Violet 785™ anti-mouse F4/80 Antibody, clone BM8, BioLegend, #123141;
PE/Dazzle™ 594 anti-mouse/rat CD61 Antibody, clone 2C9.G2, BioLegend, #104321;
PE/Cyanine7 anti-mouse CD154 Antibody, clone MR1, BioLegend, #106512;
Brilliant Violet 605™ anti-mouse CD45 Antibody, clone 30-F11, BioLegend, #103139;
Pacific Blue™ anti-mouse CD41 Antibody, clone MWReg30, BioLegend, #133932;
PE anti-mouse CD49e Antibody, clone 5H10-27(MFR5), BioLegend, #103805;
PE anti-mouse CLEC-2 (CLEC1B) Antibody, clone 17D9/CLEC-2, BioLegend, #146103;

APC anti-mouse CD14 Antibody, clone Sa14-2, BioLegend, #123312;
APC anti-mouse CD31 Antibody, clone MEC13.3, BioLegend, #102510;
rat anti-mouse CD88 (C5aR) Antibody, BioLegend #135815.

Emfret Analytics:
PE- and FITC- labeled antibodies, Clones JON/A / Wug.E9, Emfret Analytics, #D200;
FITC-labeled Rat Anti-Mouse GPVI Monoclonal Antibody, Clone JAQ1, Emfret Analytics, #M011-1;
Integrin alphaIIbbeta3 (GPIIb/IIIa, CD41/CD61), clone Leo.F2, Emfret Analytics, #M025-1;
GPIbalpha (CD42b), clone Xia.G5, Emfret Analytics, #M040-2;
Integrin alpha5 chain (CD49e), clone Tap.A12, Emfret Analytics, #M080-1;
GPIX (CD42a), clone Xia.B4, Emfret Analytics, #M051-1;
Dylight 488 conjugated CD41 antibody, Emfret Analytics, #X488;

eBioscience:
CD45 Mouse anti-Human, PE-Cyanine5.5, Clone: HI30, eBioscience, #350459-42;
CD144 (VE-cadherin) Monoclonal Antibody (eBioBV13 (BV13)), Alexa Fluor 488, eBioscience, #53-1441-82.

Invitrogen:
Isolectin GS-IB4 Alexa Fluor 594. - Isolectin GS-IB4 From Griffonia simplicifolia, Alexa Fluor™ 594 Conjugate, Invitrogen, # I21413;
donkey anti-goat Alexa Fluor 488 antibody, - Donkey anti-Goat IgG (H+L) Cross-Adsorbed Secondary Antibody, Alexa Fluor 488, Invitrogen, # A-11055;
goat anti-rat Alexa Fluor 568 antibody - Goat anti-Rat IgG (H+L) Cross-Adsorbed Secondary Antibody, Alexa Fluor 568, Invitrogen, #A11077;
goat anti-rabbit Alexa Fluor 488 antibody, # A-11008

Merck:
anti-NG2 Chondroitin Sulfate Proteoglycan Antibody, Merck, # AB5320;

Abcam:
Goat Anti-Rat IgG HRP-conjugated Antibody, (Abcam, ab205718, 1:2000)
rat anti-mouse anti-C3 antibody, clone 11H9, Abcam, ab11862, 1:20)
phospho PKA (PKA α/β/γ catalytic subunit phospho T197), Abcam, #ab75991, 1:3000),
total PKA (α/β catalytic subunits, Abcam, #ab216572, 1:500);
anti-β-actin (Abcam, #8226, 1:1000).
rat anti-mouse anti-C3 antibody (clone 11H9, ab11862, Abcam, Milton, UK, 1:20)
donkey anti-rat preadsorbed IgG Alexa Fluor 568 (ab175475, Abcam, 1:500)
donkey anti-mouse preadsorbed IgG Alexa Fluor 488 (ab150109, Abcam, 1:500)
goat anti-rabbit IgG coupled with biotin (ab6720, Abcam, 1:5000)
Goat Anti-Rabbit IgG H&L (HRP) (ab205718)
Goat Anti-Mouse IgG H&L (HRP) (ab205719)

Dianova:
Donkey F(ab’)2 anti-rat IgG (H+L)-Alexa Fluor 647, Dianova, #712-606-153;
Donkey anti-Rabbit IgG (H+L)-Cy3, Dianova, # 711-165-152;
Donkey F(ab’)2 anti-Rabbit IgG (H+L)-Alexa Fluor 647, Dianova, # 711-606-152;

LI-COR:
IRDye® 800CW Goat anti-Rat IgG Secondary Antibody, Li-COR, #926-32219;

Novus Biologicals:
anti-mouse CD63-PECy5.5, clone NKI/C3, Novus Biologicals, # NBP2-34694PECY55;

Santa Cruz:
α-actin antibody, clone1A4, Santa Cruz, # sc-32251;
goat anti-mouse C5aR1 antibody cone P14, CD88, Santa Cruz, # sc-3124
mouse monoclonal P-selectin antibody, clone CTB201, Santa Cruz, #sc-8419;
C3aR Antikörper, clone D-12, Santa Cruz, # sc-133172;

Cloud Clone Corporation:
Polyclonal Antibody to Platelet Factor 4 (PF4), Cloud Clone Corporation, # PAA172Mu01;

Sigma Aldrich:
Monoclonal anti-α-Tubulin-antibody, clone B-5-1-2, Sigma Aldrich, #T5168;

Hycult Biotech:
rat anti-CD88 antibody, clone 10/92, Hycult, # HM1077-100UG;

BD Biosciences:
rat anti-mouse anti-CD102 Clone 3C4 - Purified Rat Anti-Mouse CD102, Clone 3C4(mIC2/4), BD Biosciences, #553326;
purified rat anti-mouse CD62P - Purified Rat Anti-Mouse CD62P, Clone RB40.34, BD Biosciences, #550289;

Proteintech:
thrombospondin 1 (rabbit polyclonal Anti-Thrombospondin-1 Antibody, Proteintech, Chicago, Illinois, USA, #1 18304-1-AP;

Thermo Fisher Scientific:

Donkey anti-Rat IgG (H+L) Highly Cross-Adsorbed Secondary Antibody, Biotin, Invitrogen, # A18749;
Donkey anti-Rabbit IgG (H+L) Highly Cross-Adsorbed Secondary Antibody, Alexa Fluor 647, Invitrogen, # A-31573;
Goat anti-Rat IgG (H+L) Cross-Adsorbed Secondary Antibody, Alexa Fluor 488, Invitrogen, # A-11006;

Cell Signaling Technology:
phospho PLCγ2 (Tyr1217, Cell Signaling Technology, #3871S, 1:1000), total PLCγ2 (Cell Signaling Technology, Danvers, USA, #3872S, 1:1000);
phospho Akt (Ser473, Cell Signaling Technology, #9271S, 1:1000),
total Akt (Rabbit monoclonal Akt pan C67E7, Cell Signaling Technology, # 4691S, 1:1000);
phospho PI3K (Phospho PI3 Kinase p85 Tyr458/p55 Tyr199, Cell Signaling Technology, #4228S, 1:1000),
total PI3K (rabbit monoclonal PI3 Kinase p85 19H8, Cell Signaling Technology, #4257S, 1:1000);
phospho GSK 3β (Ser9, Cell Signaling Technology, #9336S, 1:1000),
total GSK 3β (rabbit monoclonal anti GSK 3β 27C10, Cell Signaling Technology, #9315S, 1:1000);
phospho p44/42 MAPK (Erk1/2 Thr202/Tyr204, Cell Signaling Technology, #9101S, 1:1000),
total p44/42 MAPK (Erk1/2, Cell Signaling Technology, #9102S, 1:1000);
phospho PLC β 3 (Ser537, Cell Signaling Technology, #29021S, 1:1000),
total PLC β 3 (rabbit monoclonal PLCβ3 D9D6S, Cell Signaling Technology, #14247, 1:1000);
phospho PKC (Ser) substrate (Cell Signaling Technology, #2261S, 1:1000),
total PKCα (Cell Signaling Technology, #2056S, 1:1000).

ELISAs:
mouse PF4/CXCL4 Quantikine ELISA Kit (R&D) , a membrane-based antibody array (Proteome Profiler Mouse Angiogenesis Array Kit, ARY015, R&D),
a Serotonin ELISA kit (BA E-8900, LDN, Nordhorn, Germany),
a Hexosaminidase B B (HEXb) ELISA Kit (SEA637Mu, Cloud Clone Corporation),
a Mouse VEGF ELISA Kit (ab209882, Abcam),
a Thrombospondin 1 ELISA Kit (THBS1, ABIN6574175, Antibodies-Online, Aachen, Germany),
an Endostatin COL18A1/ES ELISA kit (Mouse collagen type XVIII α 1 Endostatin ELISA Kit, MBS701673, MyBioSource.com, San Diego, USA)
and a TIMP-1 ELISA Kit (Mouse TIMP-1 Quantikine ELISA, MTM100, R&D).
human CXCL4/PF4 Quantikine ELISA kit (MCX400 ,R&D),
a human PDGF BB ELISA kit (ab100624, Abcam)

Validation

For flow cytometry experiments:
All antibodies used were recommended by the manufacturer for flow cytometry applications. The required concentration was arrived at by titration experiments with maximization of the calculated stain index. Specificity was tested both by fluorescence-minus-one controls as well as isotype control staining.
For immunofluorescence staining:
All antibodies used were recommended by the manufacturer for application in immunofluorescent staining of frozen sections. Specificity was assessed by performing staining with and without primary antibody but with secondary antibody on the same slides as a standard control. For initial testing of primary antibodies, we used isotype controls in equimolar concentrations. All secondary antibodies were approved by the manufacturer for the intended use.

Eukaryotic cell lines

Policy information about cell lines

Cell line source(s)

MHEC-5T (Leibniz Institute DSMZ-German Collection of Microorganisms and Cell Cultures), HUVECs (PromoCell, Heidelberg, Germany)

Authentication

No authentication was performed.

Mycoplasma contamination

The cell lines were not tested.

Commonly misidentified lines
(See ICLAC register)

None of the cell lines used is a commonly misidentified line.

Animals and other organisms

Policy information about studies involving animals; ARRIVE guidelines recommended for reporting animal research

Laboratory animals

A detailed description of each mouse, strain, sex and age is reported in the materials and methods section of the manuscript. Mice for hindlimb ischemia experiments were aged 10-12 weeks, mice for blood withdrawal were 6-16 weeks. Light cycle was 12 h, temperature 20-22 °C, humidity 40-60%.

Wild animals

The study did not involve wild animals

Field-collected samples

The study did not include field-collected samples

Ethics oversight

All animal procedures were approved by the regional animal care and use committee of the District of Tübingen, Baden-Württemberg (Konrad-Adenauer-Straße 20, 72072 Tübingen, Germany). All animal experiments were performed in accordance with the German law guidelines of animal care.

Note that full information on the approval of the study protocol must also be provided in the manuscript.

Human research participants

Policy information about studies involving human research participants

Population characteristics	Only very few healthy volunteers participated in the experiments reported here by donating blood for human platelets isolation.<br>All participants were free from drug usage of any kind for 2 weeks. Both females and males were included.<br>Only healthy individuals donated blood. Thus, there were no diagnoses, the mean age of research participants was 31.42 +/- 4.1 (SEM)
Recruitment	Volunteers participated freely. No further data was collected on them. As only cells were isolated from the blood of volunteers and cells from each individual were used for all groups in the subsequent experiments, self-selection bias does not apply.
Ethics oversight	All procedures were in accordance with German Federal Law and Regulations and performed under supervision of the institutional ethics committee. For experiments with human material, written informed consent was received from participants prior to inclusion in the study. The study was approved by the institutional ethics committee (270/2011BO1) and complies with the Declaration of Helsinki and the good clinical practice guidelines.

Note that full information on the approval of the study protocol must also be provided in the manuscript.

Flow Cytometry

Plots

Confirm that:

☒ The axis labels state the marker and fluorochrome used (e.g. CD4-FITC).

☒ The axis scales are clearly visible. Include numbers along axes only for bottom left plot of group (a 'group' is an analysis of identical markers).

☒ All plots are contour plots with outliers or pseudocolor plots.

☒ A numerical value for number of cells or percentage (with statistics) is provided.

Methodology

Sample preparation	The cell source and sample preparation for flow cytometry is outlined in the methods section of the manuscript in detail.<br>Murine whole blood: Blood was diluted 1:5 using Tyrode's solution (pH 7.4, supplemented with 1 mM CaCl2 and 1 mM MgCl2). In some experiments, blood was stimulated with the following agonists for 10 min at 37°C: ADP (5, 20, 100 µM, Chrono-log Corporation, Havertown, PA, USA); collagen related peptide (CRP; 0.5, 2, 5 µg/ml; CambCol Laboratories, Cambridge, UK); C5a (2, 20, 200, 2000 ng/ml; R&D). After stimulation, blood was diluted 1:5 once again to stop activation and staining was performed at room temperature for 30 minutes. After staining, cells were fixed using freshly prepared 4% PFA solution and diluted using FACS buffer (PBS, 0.5% BSA, 0.1% Na-Azide).
Instrument	A Fortessa Instrument manufactured by BD Biosciences was used for analyzing some samples. Some samples were analyzed on a FACS-Calibur flow cytometer (Becton-Dickinson, Heidelberg, Germany). Most samples were analyzed on a Cytoflex-S (4 lasers, 13 colours, Beckman Coulter).
Software	FACS Diva (BD Biosciences) software was used for data collection from all biological samples run on the Fortessa and the Calibur<br>Instrument (BD Biosciences). Data analysis was performed using FlowJo v9. For the Cytoflex, CytExpert Softwatre (v. 2. 4) was used for acquisition and analysis.
Cell population abundance	The abundance of platelets was about 7 - 10 % relative to all events in murine whole blood.<br>The abundance of single platelets in human whole blood was about 3-6 % relative to all events.<br>The puritiy of isolated platelets was over 95% both for human as well as mouse samples.
Gating strategy	Gating strategies are shown on representative flow cytometry plots where necessary.<br>Gating for mouse as well as human platelets was performed by CD41 and CD45 as well as FSC and SSC at a logarithmic scale.<br>Platelets were defined as CD45-CD41+ and differentiated from debris using FSC and SSC (see also Suppl. Fig. 26).

☒ Tick this box to confirm that a figure exemplifying the gating strategy is provided in the Supplementary Information.
